# Non-Coding RNAs as Regulators and Markers for Targeting of Breast Cancer and Cancer Stem Cells

**DOI:** 10.3390/cancers12020351

**Published:** 2020-02-04

**Authors:** Kirti S. Prabhu, Afsheen Raza, Thasni Karedath, Syed Shadab Raza, Hamna Fathima, Eiman I. Ahmed, Shilpa Kuttikrishnan, Lubna Therachiyil, Michal Kulinski, Said Dermime, Kulsoom Junejo, Martin Steinhoff, Shahab Uddin

**Affiliations:** 1 Translational Research Institute, Academic Health System, Hamad Medical Corporation, Doha 3050, Qatar; hamnafathima2001@gmail.com (H.F.); emoibrahim04@gmail.com (E.I.A.); SKuttikrishnan@hamad.qa (S.K.); LTherachiyil@hamad.qa (L.T.); MKulinski@hamad.qa (M.K.); MSteinhoff@hamad.qa (M.S.); skhan34@hamad.qa (S.U.); 2 National Center for Cancer Care and Research, Hamad Medical Corporation, Doha 3050, Qatar; ARaza@hamad.qa (A.R.); SDermime@hamad.qa (S.D.); 3 Sidra Medicine, Doha 26999, Qatar; tkaredathabdulazis@sidra.org; 4 Department of Stem Cell Biology and Regenerative Medicine, Era University, Lucknow 226003, India; drshadab@erauniversity.in; 5 Qatar College of Pharmacy, Qatar University, Doha 3050, Qatar; 6 General Surgery Department, Hamad General Hospital, Hamad Medical Corporation, Doha 3050, Qatar; KJunejo@hamad.qa; 7 Department of Dermatology Venereology, Hamad Medical Corporation, Doha 3050, Qatar; 8 Department of Dermatology, Weill Cornell Medicine, Qatar Foundation, Education City, Doha 24144, Qatar; 9 Department of Medicine, Weill Cornell Medicine, New York, NY 10065, USA

**Keywords:** breast cancer stem cells, biogenesis, long non-coding RNA, microRNA, targets

## Abstract

Breast cancer is regarded as a heterogeneous and complicated disease that remains the prime focus in the domain of public health concern. Next-generation sequencing technologies provided a new perspective dimension to non-coding RNAs, which were initially considered to be transcriptional noise or a product generated from erroneous transcription. Even though understanding of biological and molecular functions of noncoding RNA remains enigmatic, researchers have established the pivotal role of these RNAs in governing a plethora of biological phenomena that includes cancer-associated cellular processes such as proliferation, invasion, migration, apoptosis, and stemness. In addition to this, the transmission of microRNAs and long non-coding RNAs was identified as a source of communication to breast cancer cells either locally or systemically. The present review provides in-depth information with an aim at discovering the fundamental potential of non-coding RNAs, by providing knowledge of biogenesis and functional roles of micro RNA and long non-coding RNAs in breast cancer and breast cancer stem cells, as either oncogenic drivers or tumor suppressors. Furthermore, non-coding RNAs and their potential role as diagnostic and therapeutic moieties have also been summarized.

## 1. Introduction

Breast cancer (BC) is the most common form of cancer among women and accounts for 11.6% of cancer incidences and 6.6% of cancer-associated deaths worldwide [1]. The high incidence and death rates in BC are linked to various factors, among which the most common being its heterogeneous nature. The inter/intratumoral heterogeneity, usually affecting one anatomic site of the breast with phenotypic and molecular diversity, plays a key role in its histology and staging [2,3]. Previously, histological stratification of BC was based primarily on the expression status of hormonal receptors, such as the estrogen receptor (ER), progesterone receptor (PR), and ERBB2 receptor (HER2) [4]. However, with advances in molecular analysis and gene expression profiling, further subtypes of BC, including luminal ER positive (luminal A and luminal B), HER2 enriched and triple negative (basal like) have been identified [5]. This molecular sub-classification has served as a guiding principle for the utility of targeted therapies such as synthetic lethality using poly ADP ribose polymerase (PARP) inhibitors HER2-targeted (e.g., Trastuzumab) and hormonal (e.g., Tamoxifen) therapies, leading to better outcomes and management of BC [5]. Several organizations including the American Society of Clinical Oncology (ASCO) and National Comprehensive Cancer Network (NCCN) have also issued extensive recommendations and guidelines for implementation of molecular analysis as a tool for risk stratification, treatment planning and management [6,7,8]. 

Currently, the individualized treatment strategy is based on various factors including tumor size, morphology, grade, metastases, ER, PR and HER2 expression [9]. While detailed information about these factors is critical for therapeutic management, identification and understanding of these diagnostic/predictive markers will aid in implementing personalized treatment strategies. Therefore, breakthrough data on transcriptional regulators of gene expression, known as “non-coding RNA” has become a focus of research worldwide.

The transcriptome of most organisms is far more complex than originally imagined, as the vast majority of genomic sequence is extensively transcribed into a diverse range of protein coding and non-coding RNAs (ncRNAs) [10]. Surprisingly, out of 75% of the transcribed human genome, only about 2% represents the protein coding region [11]. Until recently, the majority of the transcriptome which lacks coding potential was considered to be “Junk” or products of faulty aberrant splice events [11]. Considerable improvements in high-throughput technologies, such as RNA sequencing, have allowed the identification of several previously unannotated non-protein coding transcription events in genomic regions. The efforts for re-evaluating non-coding part of the human genome and re-classifying them from “junk” to “non-junk” have been accomplished mainly through the Encyclopedia of DNA Elements project (ENCODE) project and by using ab initio transcriptome assembly which provides unbiased modality for lncRNA discovery which can pinpoint cancer- associated ncRNAs [12,13]. These projects provided critical insights into the “junk” or “dark matter” of DNA being transcribed via complex regulatory networks for the regulation of coding genes. Thus, the pinnacle of interest was shifted from coding genes to transcripts as the fundamental units of the genome. 

The classification of the non-coding part of the genome, known as ncRNAs, is based on their length. Keeping the cutoff at 200 nucleotides’ length, the ncRNAs <200 nucleotides are designated as short noncoding RNAs (sncRNAs). These include microRNA (miRNA), small interfering Ribonucleic Acid (siRNA), piwi-interacting RNA (piRNA), small nucleolar RNAs (snoRNAs), small nuclear RNA (snRNA), and tRNA-derived fragments (tRFs) [14]. The ncRNAs >200 nucleotides, known as lncRNAs [15] include intronic, antisense, long intervening/intergenic noncoding RNAs (lincRNA), competing endogenous RNA (ceRNA), etc. [16]. Both miRNAs and lncRNAs can control fundamental cellular and biological processes via diverse mechanisms and have been associated with playing key regulating roles in transcriptome by establishing networks and interactions. Since miRNAs are considered to be negative regulators of gene expression, lncRNAs are also considered to be an important regulator in different ways of gene expression including cross-talk with miRNA, sponging the microRNA, and regulating their expression [17,18,19]. The expression and function of miRNAs and lncRNAs are tightly regulated and conserved in development and physiological homeostasis. The role of miRNAs and lncRNAs is critical and leads to the pathogenesis of various human diseases such as cancer by dysregulation of human transcriptome [20]. 

**Figure 1:**
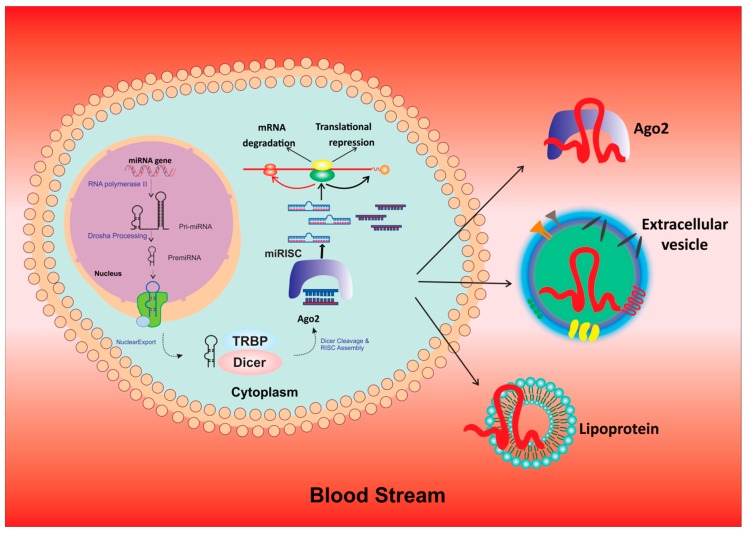
Process of biogenesis of miRNAs in the nucleus, its transfer into cytoplasm and functions.

The miRNAs are small, 18–23 nucleotide long transcripts involved in gene regulation via post-translational modifications [21]. The mechanism of action of miRNA involves interacting by binding to the open reading frame or to the 3’ untranslated regions (3′ UTRs) of target genes, which leads to repression of gene expression of the translating mRNA or mRNA degradation through formation of functional complexes via activation of Argonaute (Ago) proteins which target the 3′ UTRs [22]. The biogenesis of miRNAs is shown in detail in Figure 1. Numerous studies documented the role of miRNA in cancer progression. Oncogenic miRNAs are associated with regulation of tumor suppressor genes and targeting of oncogenes thus promoting invasion, metastasis, and drug resistance [23]. 

**Figure 2:**
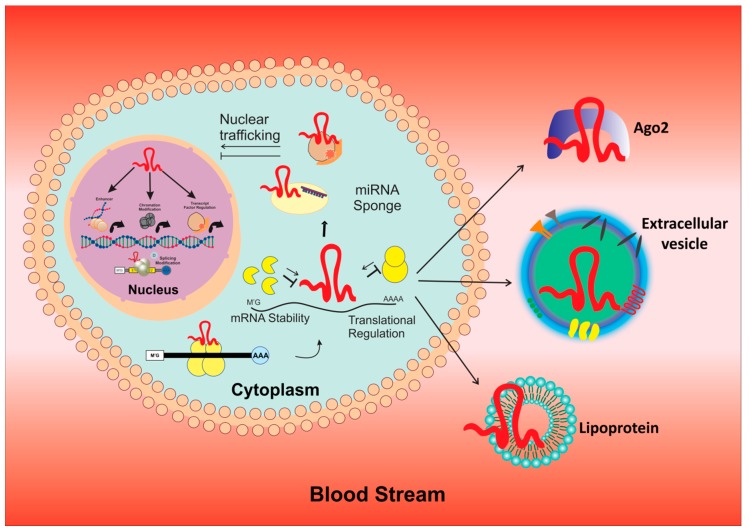
Illustrates the mechanism involved in process of biogenesis and function of lncRNA.

In addition to miRNAs, lncRNAs [24,25] were been reported for their functionally important roles in cancers [16,26]. The biogenesis of lncRNA is a complex process with capping, splicing, and polyadenylation [27,28]. The main mechanisms include cleavage by ribonuclease P (RNaseP) to generate 3′ mature ends [29], the formation of snoRNA and snoRNP complex caps at the ends, and finally special 5′- and 3′ end processing to convert it into a circular stable structure [30,31,32] (Figure 2). Recently, unique sub-nuclear structures, known as “paraspeckles”, with protein-rich nuclear organelles around a specific lncRNA scaffold, were identified during biogenesis [33]. They have been said to stimulate gene regulation through sequestration of component proteins and RNAs, with subsequent depletion in other compartments [34]. 

The ENCODE project identified more than 28,000 unique lncRNAs, most of which are still not properly annotated or identified [35]. Functional characterization of several of them is still a challenge except in the case of some classically defined important lncRNAs which are well explored, such as X inactive specific transcript (XIST; in X chromosome inactivation), oncogenic lncRNA HOX Transcript Antisense Intergenic RNA (HOTAIR); in positional identity and telomerase RNA component (TERC; in telomere elongation), ANRIL a lncRNA in molecular scaffold of chromatin-modifying complexes, decoy RNAs such as GAS5 (growth arrest specific 5) and TERRA (telomeric repeat-containing RNA) [36,37]. A plethora of regulatory functions were unveiled in several lncRNAs which affects their cellular functions associated with development and pathophysiology of diseases including several types of cancer, neurological and cardiovascular conditions, and immunological and metabolic disorders [38,39,40]. 

Published data underpinned the roles played by miRNA and lncRNA in invasion and metastasis in BC and Breast cancer stem cells (BCSCs). However, a detailed study on the interaction of ncRNA with cancer stem cells (CSCs) and their effects on metastasis and recurrence has not yet been carried out. Our present review aims to outline research studies that highlight the impact of miRNAs and lncRNAs on tumor occurrence and progression in BC and BCSCs, while also underscoring the potential role governed by ncRNAs as diagnostic and therapeutic moiety that may lay as future foundation in development of newer strategies to prevent and overcome issues related to invasion and metastasis in BC and BCSCs. 

## 2. BCSCs and Their Regulation 

CSC is a small population that exhibits characteristics of both cancer cells and stem cells including self-renewal, differentiation, asymmetric/symmetric division, as well as alterations in their gene expression. CSCs have the ability to seed tumors when transplanted into an animal host as well as give rise to non-CSC bulk tumors in order to promote disease progression [41,42]. Therefore, BCSCs represent a heterogeneous population of cancer cells that possess the ability to form transplantable tumors, tumor maintenance, progression, therapeutic resistance, and relapse [43]. Characterization of BCSC has shown that they express a panel of markers depending on their source of derivation. For example, when isolated from transgenic mouse models, BCSC tend to express CD133^+^, CD24^+^ Thy1^+^, CD29^lo^ CD24^+^ CD61^+^, Sca1^+^, CD24^+^ CD29^+^/CD49f^+^ whereas when isolated from cell lines, the main markers for identification include MUC1^+^, Procr^+^/ESA^+^, CD49f^+^/DLL1^hi^/DNER^hi^, GD2^+^, CD44^+^/CD24^−^/^lo^/ANTXR1^+^, ABCG2^+^, Lgr5^hi^, CD44^+^CD24^-^/^lo^SSEA-3^+^ or ESA^hi^PROCR^hi^SSEA-3^+^, Nectin-4^+^ and CD70^+^ [44]. However, the most widely used markers for identification are CD44/CD24 and ALDH1 [45]. It has been reported that tumors expressing even a small number of cells with CD24^−^/CD44^+^ and ALDH1^+^ markers exhibit an increased tumor-initiating capacity in NOD/SCID mice [46] indicating the significance of these two distinct subtypes in BCSC characterization especially with respect to their location and proliferation capability [45]. In BC, mesenchymal-epithelial transition (MET) CSCs bears higher ALDH expression as well as higher proliferation rate is contrary to epithelial-mesenchymal transition (EMT) CSCs which are enriched with CD44^high^/CD24^-^ expression but with poor proliferation rate. However, aggressive clinical behavior in tumor types is attributed to the high proportion of ALDH-expressing CSCs [45,47]. 

## 3. BCSCs and Tumor Microenvironment

The normal breast tissue is highly heterogeneous and has the unique capacity to self-renew/regenerate, proliferate and differentiate into mature luminal and myoepithelial cells with the help of mammary stem cells (MaSCs) that reside within the microenvironment [48,49]. The regulation of these MaSCs is dependent upon the components of the microenvironment including blood vessels, immune cells, signaling molecules, fibroblasts, and the extracellular matrix (ECM) [48,50,51]. Similarly, in BC, the tumor microenvironment (TME), consisting of cancer-associated fibroblasts (CAFs), MSCs, immune cells, immune-suppressive cells, endothelial cells, cytokines, growth factors, etc. are known to play a critical role in the regulation and modulation of BCSCs thus facilitating therapeutic resistance, metastasis, and progression [52]. 

The role of various components of the TME in BCSCs activity is documented in several studies [53,54,55]. For example, CAFs within the microenvironment release several growth factors, hormones like platelet-derived growth factor-BB, cytokines, and chemokines, such as CCL2, CCL7, IL-6 and IL-8, that modulate CAFs and promote stemness and expansion of BCSC [55,56,57,58,59]. CAFs are considered to be a central core component in the maintenance of CSC properties thereby promoting stemness in BC cells [60,61,62,63]. Similar to CAFs, another important component of the tumor stroma that plays a role in the expansion of BCSCs is MSCs [53]. Studies reported that MSCs regulate increased production of CXCL7 and IL-6 via positive feedback mechanism that promotes BCSC self-renewal, expansion as well as metastatic potential [64].

In addition to CAFs and MSCs, a variety of immune cells including T cells, macrophages, and T regulatory cells (Tregs) also play a critical role in the modulation of TME to promote the expansion of BCSCs [65]. In the past, several studies have reported that tumor-associated macrophages (TAMs) are commonly involved in the expansion of BCSCs via the up-regulation of HAS2 (hyaluronan synthase) and paracrine EGFR/STAT3/SOX-2 signaling pathway [66,67]. In addition to this, TAMs promote the secretion of cytokines including IL-6, IL-8, GM-CSF, TNF-α and TGF-β that allows regulation, maintenance, and proliferation of BCSCs [52,68]. 

## 4. Regulatory Pathways Associated with BCSC

The regulation of BCSCs is largely dependent on key signaling pathways including JAK/STAT, Notch, Wnt, and Hedgehog [69,70,71,72]. The dysregulation of these pathways facilitate differentiation and self-renewal of BCSCs leading to increased proliferation, invasion, and metastasis in cancers [69,73].

Accumulating evidence suggests that dysregulation of the JAK/STAT3 pathway is the common mechanism involved in the maintenance/regulation of BCSCs [74,75]. In BC, the modulation of TME via secretion of cytokines, growth/transcription factors including IL6/STAT3, NO/NOTCH, Twist2 and hormones such as leptin facilitate activation/phosphorylation of JAK/STAT3 pathways leading to enhanced self-renewal and differentiation capacity in BCSCs [76,77,78]. In addition to this, studies have reported that the activation of JAK/STAT3-Regulated Fatty Acid β-Oxidation I (STAT3-CPTIB-FAO) and EGFR/STAT3/SOX-2 paracrine signaling also play an important role in conferring drug resistance -associated characteristics to BCSCs thus leading to treatment failures [66,79]. Another signaling pathway that is known to be involved in the maintenance and self-renewal of BCSCs is the Notch signaling pathway [69,70]. This pathway is activated via binding of Notch receptors to Notch ligands thus leading to translocation of the Notch intracellular domain (NCID) to the nucleus. The subsequent hyperactivation of downstream effector molecules regulates the asymmetric division and self-renewal of BCSCs [69,70]. Increased levels of Notch1 are associated with increased ALDH1 levels in BCSCs indicating that Notch signaling dysregulation is important for BCSC proliferation and maintenance [80]. Reports also suggest that the expansion of BCSCs is influenced by several factors such as histone-lysine N-methyltransferase (Enhancer of Zeste Homolog 2; EZH2) and lipid mediator sphingosine-1-phosphate (S1P). Increased levels of EZH2 and SIP enhance NOTCH1 activation and signaling leads to increased tumorigenic ability in mice and breast cancer patient- derived mammospheres [81,82].

The Wnt/Frizzled/-catenin signaling is a critical pathway that activates Wnt-targeted transcription factors via nuclear translocation of cytosolic b-catenin. This, in turn, facilitates activation of Wnt-targeted genes through binding to the T cell factor/lymphoid enhancing factor family (TCF/LEF) leading to activation of genes associated with cellular differentiation, asymmetric division and cell migration [74,83]. In BCSCs, activation of Wnt signaling due to transcription factor Sry-related HMG box 9 (Sox9) supported stemness and increased mammosphere-formation in BC cell lines thus suggesting that increased Wnt signaling is associated with enhanced BCSC proliferation, self-renewal, and maintenance [84]. 

The Hedgehog pathway is also an important signaling pathway that is activated via smoothened that facilitates cytoplasmic translocation of Gli-com to the nucleus [69]. Like Wnt signaling pathway aberrant activation of Hedgehog pathway due to overexpression of smoothened or due to various growth factors (fibroblast growth factor 5 (FGF5) and collagen), EMT, MET, CAF have been observed to be involved in maintenance, proliferation and, stemness of BCSCs [60,61,65,85,86,87] Therefore, the Hedgehog pathway is considered to be an important regulatory pathway for maintenance of stemness in breast cancer cells [69]. 

## 5. Role of MicroRNAs and LncRNA in BCSCs

MicroRNAs, including oncomiRs and Tsmirs, have been critically implicated in the regulation of BC development and progression via regulatory networks. Modulation of signaling pathways such as PI3 kinases, Wnt/βcatenin, STAT, HIF 1α, etc. by miRNAs directly or indirectly influences hallmarks of cancers and facilitates tumor suppression/progression [88]. Studies have shown that functional interaction of miRNA with cell proliferation and cell cycle progression factors such as cyclin protein families, protein kinases, etc. serves as an important target for tumor suppression/proliferation in BC [88]. For example, miRNAs, such as miR-497, miR-16, and miR-30c-2-3p, were reported to target and inhibit cell cycle regulator of G1-S transition, cyclin E1 leading to decreased cyclin E1 expression and suppression of proliferation by blocking BC cells from entering the S-phase of the cell cycle [89,90,91,92]. On the other hand, certain miRNAs, such as miR-483-3p, dysregulate the cell cycle transition by facilitating the formation of cyclin E1 and cyclin-dependent kinase CDK2 complex. This leads to increased expression of cyclins, up-regulation of protein kinases and down-regulation of kinase inhibitors, thereby increasing BC cell viability and proliferation [92]. Similarly, overexpression of miR-1207-5p, has been associated with negative regulation of STAT2 expression and inactivation of cell cycle-dependent kinase inhibitors CDKN1A and CDKN1B thus promoting cell cycle progression in cancer cells [93].

The WNT/β catenin pathway is a well-documented target of miRNAs. Various studies have shown that modulation of this pathway can affect the migration/invasive potential of BC cells [88]. For example, overexpression of miR-148a has been reported to decrease migration of BC cells via targeting of WNT-1 ligand of the WNT/β catenin pathway. This leads to reduced levels of WNT-1 mRNA/protein, catenin, MMP-7, and TCF-4 levels, thus affecting the migration of cancer cells [94,95]). miR-340, has also been identified as a regulator of the WNT/β catenin pathway and acts to influence migration/invasion of BC cells via molecular targeting of associated genes such as c-MYC, CTNNB1and ROCK1 [95]. Furthermore, other signaling molecules, suppressed by miRNAs, include SMAD7, MTA1, WT1, SETBP1, EphA4, LASP1, and STAT3. Suppression of these molecules via down-regulation of miRNAs including miR-497, miR-421, miR-193a etc. leads to reduced migration/invasion potential of BC cells [96,97,98].

In addition to the regulation of the Wnt/β catenin pathway, certain miRs have also been identified to regulate the PI3K/Akt signaling pathway [99,100]. For example, miR-204-5p is important in BC as its overexpression leads to a reduction in cell proliferation, migration, and metastasis via direct inhibition of PIK3CB. Furthermore, it is also involved in modulation of key immune cells such as myeloid-derived suppressor cells (MDSCs), macrophages, and natural killer (NK) cells to supports cancer cell proliferation via remodeling of tumor microenvironment [101]. 

**Figure 3:**
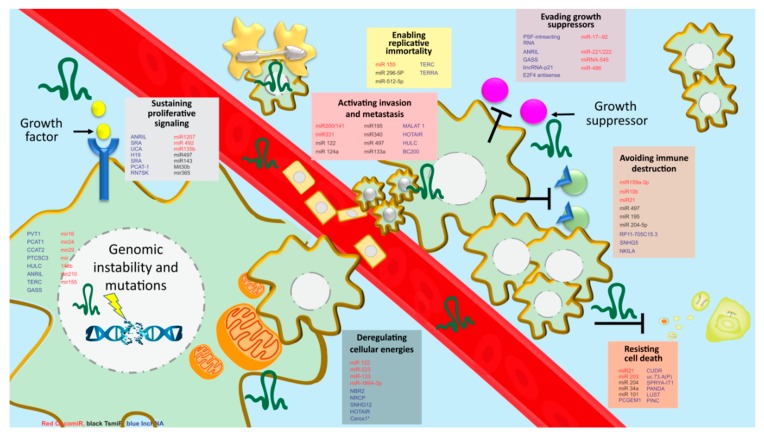
MicroRNA and LncRNA involved in breast cancer stemness therapy resistance and tumorigenesis. There are eight hallmarks implicated in cancer including sustaining proliferative signaling, enabling replicative immortality, evading growth suppressors, activating invasion and metastasis avoiding immune destruction, resisting cell death, deregulating cellular energetics and genomic instability and mutations. Expression of several microRNAs and lncRNA is associated with inducing oncogenic or tumor-suppressive properties via using the hallmarks of cancers.

Like in BC cells, miRNAs are associated with directing their oncogenic/suppressor potential in BCSCs (Figure 3, Table 1) [102]. For example., miR-200 family comprising of miR-200a, miR-200b and miR-200c [103] is well-known for their extensive role in conferring stem cell-like properties in BC cells including mammospheres formation, EMT regulation, metastasis, invasion, apoptosis, survival, and cancer cell growth [103,104]. There are various mechanisms by which miR-200b and miR-200c modulate target genes in order to facilitate stem cell-like properties. For instance stem cell transcription factor KLF4, suppressor of zeste 12 (SUZ12), poly-comb complex protein BMI1 and Prolyl isomerase Pin1 are frequently targeted by miR-200c leading to transcription repression and influencing BCSC formation [105,106,107]. On the other hand, the up-regulation of miR-200 decreases the expression of ZEB1/ZEB2 leading to reduced expression of E-cadherin and affecting the metastatic potential of BCSCs [108,109]. Similarly, studies documented that increased expression of miR-200c via direct binding of tumor suppressor tumor protein p53 (p53) leads to decreased stem cell properties in BC [110]. Furthermore, knockdown of miR-200 was reported to promote mammosphere-formation via direct targeting of the ten-eleven translocation (TET) family and leading to enhanced metastasis in a mouse xenograft model [111]. In addition to this, EGF-driven invasion was also reported to be regulated and controlled by the miR-200 family [104]. 

Another miRNA family that plays an important role in BCSCs is the miR-34 family. Studies have shown that miR-34 family members, usually activated by p53 [182], are well-known to influence CSC such as properties in BC [165,183,184]. Their mechanism of action is via meditation of cell cycle arrest/apoptosis [182] as well as targeting of various signaling pathways such as BCL-2, CCND1 MYC, E2F3 CDK6, SIRT1, and Notch1/4 leading to negative regulation of cell proliferation, invasion, migration, and subsequent inhibition of BCSCs propagation [183,185,186,187]. Similarly, a study on BC patient tissues has shown that miR-34 is negatively correlated with tumor stages and metastasis indicating its role in breast cancer progression [188]. Furthermore, overexpression of miR-34a and miR-34c has been documented to reduce mammospheres formation, inhibit the development of CD44^+^CD24^-^/ALDH^+^ cells as well as eradicate BCSCs [183,184,188].

Guarnieri et al., has reported on a novel mechanism of the miR-106b-25 cluster as a regulator of breast tumor initiation and BCSC phenotypes [189]. The results of the study show that overexpression of miR-106b-25 cluster targets repression of NEDD4L thus leading to increased NOTCH1 signaling and enhanced stem cell phenotypes in tumor imitating cells both vitro and in vivo. These results were further validated in metastatic breast cancer patient samples [189]. Similarly, the overexpression of the miR-125 family has also been associated with the modulation of stem cell-like properties in BC via targeting of receptor tyrosine-protein kinase 2/3 and Eukaryotic Translation Initiation Factor 4E Binding Protein 1 (ErbB2/3and EIF4EBP1) [190]. Overexpression of miR-125 enhances BC progression by increasing the expression of oncogenes. Therefore, miR-125 families are considered to be potential therapeutic targets [103]. Overexpression of miR-181family members via different molecular mechanisms have been associated with facilitating BCSCs in mammospheres formation, self-renewal, colony formation, tumor development as well as with poor prognosis in TNBC patients [117,189,190,191,192]. Additionally, inhibition of miR-181a/b via targeting of the Pleckstrin homology-like domain, family A, member1 (PHLDA1) has demonstrated a reduction in mammospheres formation in BC cells [193]. Furthermore, miR-27 is reported to be an important regulator of BCSCs and functions via targeting various immune mechanisms. The main mechanisms influenced by miR-27 are regulation of macrophages, activation of NF-kappaB /MAPK pathways and reduced dendritic cell-mediated differentiation of Th1 and Th17 cells [194,195]. This was shown in BC patients wherein a decrease in the miR-23a/27a/24-2 cluster in TAMs enhanced tumor growth and vice versa [196]. In addition to this, RUNX1 mediated transcriptional up-regulation of miR-27a is associated with differentiation of BCSC into endothelial cells and targeting of signaling pathways ZBTB10, MYT-1. This was reported to play a significant role in modulation of proliferation, self-renewal ability, angiogenesis, metastasis and enhanced tumorigenicity in BC cells [197].

There are a vast number of miRNAs that have been reported to be involved in the regulation of BCSCs via targeting various pathways. In addition to the ones discussed above, some of the important ones also include miR888, miR-30 family, miR-16, Let-7 family, miR-140-5p, miR-205, miR-495, etc. Overexpression or inhibition of such miRNAs can regulate the expansion of BCSCs, conversion from non-stem to stem cell phenotype, self-renewal, promotion of colony formation and affecting the number and size of mammospheres [165].

The human genome comprises 17,910 lncRNA that are often overexpressed or down-regulated in BC at various levels [198,199]. Some of the lncRNAs found to be associated with initiation, progression, and metastasis in BC include HOTAIR, Small nucleolar RNA host gene 12 (SNHG12), Long intergenic non-coding RNA for kinase activation (LINK-A), Rhabdomyosarcoma 2-associated transcript (RMST), RMRP (RNA component of mitochondrial RNA processing endoribonuclease), nuclear paraspeckle assembly transcript 1 (NEAT1), steroid receptor RNA activator (SnaR), MALAT1 (metastasis-associated lung adenocarcinoma transcript 1), CCAT2 (Colon Cancer Associated Transcript 2), CRNDE (colorectal neoplasia differentially expressed), MIAT (myocardial infarction associated transcript), MEG3 (Maternally Expressed 3), CAT104, LINC01234, STXBP5-AS1, RMRP, GATA3-AS1, RP11-279F6, AC017048 and LINC-ROR. [199,200,201,202].

In CSCs, several lncRNA such as ROR, HOTAIR, H19, UCA1, and ARSR were reported to play a significant role in stemness, proliferation, invasion, and migration via targeting of signaling pathways/sponging of various microRNA through competing for endogenous RNA (ceRNA) [199,203]. For e.g., lncRNA CRNDE was reported to be up-regulated via sponging and subsequent repression of miR-136 expression in BC cell line, MDA-MB231 as well as in BC tissues [204]. The study observed that CRNDE overexpression was associated with activation of Wnt/β-catenin, c-myc and cyclinD1 signaling pathways thus facilitating stemness, cell proliferation, migration, and invasion. Similarly, overexpression of CRNDE in mouse models showed an increase in tumor weight and volume indicating its role in promoting tumorigenesis [204]. 

lncRNA HOTAIR is a well-studied lncRNA and is reported to manifest carcinogenic potential such as migration, metastasis, invasion, EMT transition, and stemness in cancerous cells mainly via regulation of gene silencing [201]. Mir-7 by targeting the SETDB domain inhibited cellular processes, decreased the population of BCSCs and also partially reversed EMT through suppression of the STAT3 pathway in MCF-7, MDA-MB-231 cell lines and in BCSC xenograft model [205]. Furthermore, a study on CSCs of MCF-7 and MDA-MB-231 reported that HOTAIR influences migration, self-renewal, and colony formation in BCSCs via transcriptional inhibition of miR-34a and subsequent up-regulation of SOX 2. The authors validated the association of HOTAIR on functional regulation of miR-34a in BCSCs by introducing miR-34a mimics plus HOTAIR in CSCs. The results showed reduced proliferation potential of HOTAIR, thus evidencing the link between miR-34a and HOTAIR in BCSCs self-renewal and proliferative ability. On the other hand, modulation of full length HOTAIR expression was found to be associated with negative regulation of miR-34a indicating that full length HOTAIR is essentially required to affect miR-34a regulation, self-renewal, and colony formation capacity in BCSCs. In addition, up-regulated HOTAIR was also found to be involved in p53 induction thus affecting proliferation and colony formation in CSCs [206].

Another lncRNA known as lncRNAH19 is reported to be essentially involved in the induction of BC cell stemness, migration and mammosphere-formation. It functions mainly by acting as a ceRNA for miR-let 7 with subsequent overexpression of LIN28, HIF 1α, and PDK1. Since these markers are involved in inducing stem-like phenotypes, their role in BCSCs is deemed critically important. Studies on BC tissues and samples have also reported on high levels of lncRNAH19 and investigation on knockdown of H19 in nude mice has evidenced suppression of tumor growth indicating the significance of lncRNAH19 in BC tumorigenesis [207,208]. Similarly, LINC00511, a ceRNA for mir185-3p, has also been associated with influencing stemness in BCSCs. It functions by targeting E2F1 protein which in turn binds to Nanog promotor, thus forming a LINC00511/miR-185-3p/E2F1/Nanog axis leading to maintenance of BCSCs, enhanced mammosphere-formation and promotion of cell proliferation and invasion [209]. 

The TME plays an influential role in the induction of stem cell-like properties in BC cells through lncRNA. In TNBC, MSC and CAF trigger up-regulation of LINC01133 thereby inducing signaling of pluripotency factor Kruppel-Like Factor 4 (KLF4) and promoting CSC like phenotypic properties in BC cells [210]. LINC00284, another important lncRNA in TNBC has recently been identified as non-coding RNA in the aldehyde dehydrogenase 1A pathway (NRAD1) and has been documented to be functionally associated with CSCs in TNBC. This functional association and significance are based on two observations; firstly, it has been found to have genomic interactions (in the intronic regions) and secondly it is directly regulated by CSC marker ALDH1A3. This strong association indicates that NRAD1 is an important mediator of breast cancer cell proliferation and survival [211].

LncRNA RoR (regulator of reprogramming) is considered to be an important regulator of pluripotent stem cells via targeting of transcription factors SOX2, OCT4, NANOG and sponging of miR-145 [212,213,214]. As a ceRNA of mir-145, ROR functions via loss of mature miR-141 expression leading to the protection of pluripotency factors [213]. In BC cells and in patient samples, lncRNA ROR was not only linked to the self-renewal of stem cells, EMT transition, and drug resistance but also to poor prognosis indicating its significance in tumorigenesis process [215,216,217,218,219,220]. Mainly, lnc-ROR functions via targeting of ZEB1/2 and TGF-β signaling leading to modulation of EMT markers such as vimentin and neural (N)-cadherin and induction of EMT process [218,219,221,222]. Furthermore, studies on silencing/knockdown of lnc-ROR have confirmed this pathway showing that its inhibition is shown by suppression of invasiveness, migration, reduction in tumor size and reversion of drug resistance in BC cells [218,221,222]. However, its role in BCSCs and metastasis is unclear.

In addition to these, various lncRNAs such as LUCAT1, lncRNA-Hh, FGF13-AS1, lncRNA ES1 NEAT1 have been reported to be commonly involved in up-regulation of signaling pathways and modulation of stem cell factors (Wnt/β-catenin, Hedgehog, myc, SOX2, OCT4, KLF4, and NANOG). Their role in the promotion of stemness in BC cells and subsequent tumor progression, invasion and metastasis is critical for tumor maintenance and therapeutics [223,224,225,226,227].

The detailed role of lncRNAs in BC is described in Table 2.

## 6. Exosomal miRNAs: A Future Tool for Prognosis, Drug Discovery and As Therapeutic Targets

The significant presence of miRNAs was detected in biological fluids. miRNAs isolated from these sources are highly stable and non-degradable in extreme physiological conditions. It was reported that cells in culture transport intracellular miRNAs into the extracellular environment by exosomes [240]. Several studies revealed that these exosomal miRNA are implicated in cancer research, as tumor cells secrete different microRNAs capable of initiating cross-talk with the adjacent tumor microenvironment and educate them for adapting tumor favoring conditions for cancer progression [129,241,242,243,244,245]. Many exosomal miRNA were intensively studied for their ability to promote tumor progression by indicating drug resistance (miR-9,mir 221/222,miR 1246),metabolic reprogramming in CAF cells(miR105), intimating angiogenesis in endothelial cells(miR105, miR210), tumorigenesis in epithelial cells (miR10b, miR10a, miR21), osteogenesis in MSCs(mir940) [246,247,248,249]. Moreover, these exosomal miRNA can be circulated and used as potential diagnostic and prognostic markers in breast cancer [246,250]. For example, plasma and serum samples of breast cancer patients show microRNAs such as miR-106a-3p, 106a-5p, 20b-5p, and 92a-2-5p (plasma miRNAs); miR-106a-5p, 19b-3p, and 92a-3p (serum miRNAs) can be used as potential biomarkers in BC patients [251]. Some exosomal miRNA can be used as promising diagnostic markers, for example, high level of mir373 is associated with aggressive cancers, and a lower level of miR130-3p is associated with the advanced stage of cancer [252,253]. On the other hand, anticancer drugs derived from either natural or synthetic sources are reported to be dependent on miRNAs as exosomal cargoes to exert its anticancer activity [246]. For example, reduction in the growth of BC cells was associated with inhibition of secretion of exosomes containing miR-130a and miR-125 by D-rhamnose β-hederin, an oleanane type triterpenoid saponin [254]. Epigallocatechin gallate, one of the constituents present in green tea, induced its anti-cancer activity by up-regulating miR-16 in 4T1BC cells. [255] Chemosusceptibility was found to be elevated by β elemene by affecting the expression of miR-34a, miR-452, PTEN [256]. Shikonin a well-known natural compound exhibited antiproliferative effect by attenuating tumor-derived exosomal miR-128[257]. Docosahexanoic acid administration altered BC cells exosome secretion and microRNA content thereby inhibiting angiogenesis process [258]. 

Substantial evidence shows that exosomes act as a carrier and they could be manipulated to deliver tumor suppressor miRNA to exhibit their therapeutic potential [246]. Published studies have showcased that mesenchymal derived extravesicular vesicles can be successfully modified as a carrier for antitumor agents, to treat different forms of tumors [259]. The engineering of tumor-derived exosomes by electroporation method can help in overexpressing miR-155, -142, and let-7i, to mature dendritic cells and also to trigger the immunity process, to load siRNAs or miRNAs by sonication and also to knockdown oncogene such as HER2 [246]. Transfection of mesenchymal stem cells with anti-miR-222/223 transformed mesenchymal cells to dormant cancer cells and prolonged survival rate [260]. Gold-nanoparticle-facilitated RAB27A silencing in BC cells results in decreased exosomes secretion with no effect on cell viability. Exosomes were also reported to prevent tumor development both in vivo and in vitro [261,262]. Although some progress has been made to identify the potential of exosomal miRs in cancer research, it remains inconclusive as there is no standard technique reliable to isolate exosomes. The biomarker and drug therapy discoveries demand more detailed research in the field of exosomal micro RNA identification and classification.

In addition to the above techniques, using nanoparticles has also shown to increase stability and improved the delivery capability of miRNA. BC cell migration and invasion were inhibited by poly lysine-anti-miR10b complex [263]. Similarly, reduction in tumor growth capacity was observed when antisense miR-21 and antisense miR-10b were complexed with PLGA-b-PEG nanoparticle [264]. Encapsulation of miR34a with doxorubicin into hyaluronic acid chitosan successfully inhibited the migration of BC cells via the Notch-1 signaling pathway [265]. Designing various forms of nanoparticles such as gold, nano complex, and poly sorbitol-mediated transporter to carry the various form of miRNA not only improved delivery but also targeted and controlled cell proliferation of BC cells [165,266,267]. Cell cycle targeting miRNAs, miR-193a-3p and miR-214-5p encapsulated as nanoparticle showed high therapeutic potential against TNBC in vivo [268]. 

In light of the clinical impact, several miRNA-based therapies are under development whereas several of them are under pre-clinical and clinical stages. miRNA for treatment of pathologic fibrosis and blood cancer, non-small cell lung cancer and hepatocellular carcinoma is in the clinical stage however, not many lead molecules have been able to find their place either in pre-clinical or clinical trials for BC therapy. Looking at the potential of ncRNA targeting, we can assume that in the near future, the use of miRNA or lncRNA as mimics or inhibitor will be a suitable choice either alone or as an adjustment with existing therapeutic agents for regulating different aspects of human cancer [165].

In addition to the above therapies, the use of hormone therapy also known as endocrine therapy is considered to be a viable approach in point with detectable ER expression. The standard approach for treatment includes the use of tamoxifen for 5–10 years in pre-menopausal and a combination of tamoxifen with aromatase inhibition for post-menopausal women. Continuous use of tamoxifen is associated with the development of resistance; a newer viable strategy to overcome this issue is still underway [165].

The role of ncRNA in regulations of gene expression and BC implies it to be a potential target for treatment. However, data on ncRNA is still at its infancy stage with limited knowledge of its biological functions. Therefore, extensive research is required to understand its role as a prognostic, diagnostic or therapeutic target. 

## 7. Conclusions

Our review article has provided reports on extensive investigations and studies on the biological and functional role of miRNA and lncRNA in BC and CSCs providing an insight into their significance in cancer proliferation, pathological manifestations, progression invasion, and metastasis as biomarkers and as a potential therapeutic target. However, there are various considerations and challenges that need to be addressed. Firstly, in vivo studies, investigating the role of miRNAs in transgenic and knockout models are required to further ascertain their role in therapeutic targeting for the management of BC. Secondly, targeting breast cancer stem cells is a challenge in itself as accurate identification of reliable CSC markers as well as inherent heterogeneity of these cells hinders the targeting of signaling pathways by ncRNAs. Furthermore, knowledge of the types of lncRNA and their pathways in BC is still limited and extensive research to decipher its role as a biomarker/therapeutic targeting is needed. Therefore, large scale studies focusing on translational aspects of ncRNAs are required in order to fully understand and use its potential in BC treatment. 

## Figures and Tables

**Table 1:** Role of miRNAs acting as tumor suppressor/oncomir in BC with their targeted pathways.

miRNA	Type	Expression Level	Targets	Pathways	Reference
miR-31	TsmiR	↑/↓	ITGA5, RDX, RHOA	Metastasis	[112,113]
miR-145	TsmiR	↓	MUC1, ERA, RTKN	Proliferation, Apoptosis, Invasion	[114,115,116]
miR-155	TsmiR	↑	FOXO3A, RHOA, SOCS1	STAT3, Proliferation, TGFβ Signaling	[117,118,119]
miR-21	OncomiR	↑	BCL2, PTEN, MMP3, TPM1, MASPIN, PDCD4, RHOB	EMT, Apoptosis, Invasion, Migration, Inflammatory Signals	[120,121,122,123,124]
miR-125b	TsmiR	↑/↓	BAK1, ERA, HER2, CRAF, RTKN, MUC1	Migration, Proliferation, Apoptosis	[125,126,127]
miR-10b	OncomiR	↑/↓	HDAC4, TIAM, HOXD10, EMT	EMT, Metastasis, Invasion	[128,129,130]
miR-205	TsmiR	↓	HER3, VEGFA, EMT	Proliferation, Invasion	[131,132,133]
miR-210	OncomiR	↑	MNT, RAD52	Hypoxia	[134,135]
miR-196A	OncomiR	↑	ANXA1	Proliferation, Apoptosis,	[136]
miR-944	OncomiR	↑	BNIP3	Cell Proliferation, Migration, Invasion	[137]
miR-222	OncomiR	↑	PTEN	PTEN, Akt/FOXP1	[138]
miR-3646	OncomiR	↑	GSK-3β	β Catenin	[139]
miR-34A	OncomiR	↑	BCL2, CCND1	Apoptosis	[140]
miR-141	OncomiR	↑	EIF4E	Apoptosis	[141]
miR-520h	OncomiR	↑	DAPK2	PI3K/Akt	[142]
miR-34	TsmiR	↓	BCL2, NOTCH	Apoptosis, NOTCH	[143]
miR-146	TsmiR	↓	NFkB	Inflammatory Signals	[144]
miR-7	TsmiR	↓	EGFR	EGFR	[145]
miR-22	TsmiR	↓	HER3, CDK6, ERα, CDC25C, SP1	Estrogen Signaling	[146]
miR-221	TsmiR	↑	P27, P57	Wnt/β-catenin	[147]
miR-191	OncomiR	↑	SATB1, CDK6, BDNF	Estrogen Signaling	[148]
miR-196A	OncomiR	↑	ANXA1	Apoptosis	[136]
miR-335	TsmiR	↑	SOX4, TNC, PTPRN2, MERTK	Metastasis	[149]
miR-20	OncomiR	↑	E2F	Proliferation	[150]
miR-9	TsmiR	↑	LIFR, E-CADHERIN	EMT, Hippo-YAP	[151,152]
miR-126	TsmiR	↓	VEGFA and PIK3R2	VEGF/PI3K/AKT	[153]
miR-98	TsmiR	↑	ALK4 and MMP11	Angiogenesis, Invasion	[154]
miR-148a/152	TsmiR	↓	DNMT1, IGF-IR and IRS1	IGF-IR/PKM2	[155]
miR-519c	TsmiR	↓	HIF-1α	Hypoxia	[156]
miR-10b	OncomiR	↑	HOXD10	Hox pathway	[157]
miR-140-5p	TsmiR	↓	VEGFA	Metastasis, Angiogenesis	[158]
miR-494	TsmiR	↑	PTEN	Akt, NF-kB, mTOR	[159]
miR-206	TsmiR	↓	VEGF, MAPK3, and SOX9	Invasion, Angiogenesis	[160]
miR-19a	OncomiR	↑	PTEN	Oncogenic PTEN Cell proliferation, Th1 immune response	[161]
miR-17-92	TsmiR	↓	HIF-1α	Hypoxia, Angiogenesis.	[162]
miR-467	OncomiR	↑	TSP-1	Angiogenesis	[163,164]
miR-18	OncomiR	↑	SMAD7	EMT, Metastasis	[165]
miR-143	OncomiR	↑	FOSL2	EMT, Metastasis	[165]
miR-196B	OncomiR	↑	HOXD10	Hox pathway	[157]
miR-200	OncomiR	↑	ZEB1, ZEB2	EMT	[165]
miR-205	TsmiR	↓	YAP1	miR-205/YAP1, Angiogenesis, Metastasis	[166]
miR-892b	TsmiR	↑	TRAF2, TAK1, and TAB3	NF-kB	[167]
miR-210 RAD52	OncomiR	↑	RAD52	Invasion, Proliferation, Migration	[168]
mirR-155	OncomiR	↑	SOC6	STAT3 signaling	[169]
miR-451	OncomiR	↑	Bcl-2	Apoptosis	[170]
miR-100	OncomiR	↑	mTOR	Cell proliferation, Survival	[171]
miR-139-5p	OncomiR	↑	Notch1	Cell growth, Apoptosis	[172]
miR-214	OncomiR	↑	UCP2	Autophagy	[173]
miR-16	OncomiR	↑	CCNJ, FUBP1	PI3K/Akt	[174]
miR-199a-3p	TsmiR	↑	TFAM	Mitochondrial Biogenesis	[175]
miR-302b	TsmiR	↑	E2F1	E2f1-ATM axis	[176]
miR-218	TsmiR	↑	BRCA1	DNA repair, Cell proliferation, Invasion	[177]
miR-638	TsmiR	↑	BRCA1	DNA repair, Cell proliferation, Invasion	[178]
miR-29A	OncomiR	↑	PTEN	Apoptosis	[179]
miR-129-3p	OncomiR	↑	CP110	Apoptosis \, Cell Cycle, Cell Proliferation	[180]
miR-19	OncomiR	↓	Tissue factor	Angiogenesis, Metastasis	[181]

**Table 2:** Role of lncRNAs acting as either tumor suppressor/oncogene in BC with their targeted pathways.

lncRNA	Type	Expression Level	Targets	Pathways	Reference PMID
MEG 3	Tumor suppressor	↓	p53	p53	[228]
HOTAIR	Oncogene	↑	BRCA1, PTEN	PI3K/AKT-BAD pathway, HOXD10	[229]
ACNR	Tumor suppressor	↓	TGF-β	Metastasis, Invasion	[230]
PTENP1	Tumor suppressor	↓	PTEN	Apoptosis	[228]
NKILA	Oncogene	↓	NF-kB	EMT	[231]
EPIC 1	Oncogene	↑	Myc	Cell Cycle	[232]
PLNCRNA-1	Oncogene	↓	TGF-β	Apoptosis, Metastasis, Invasion	[228]
H19	Oncogene	↑	C-myc	AKT, BIK	[233,234]
MALAT-1	Oncogene	↑/↓	AKT, p53	APOPTOSIS	[235]
LINK-A	Oncogene	↑	HIF-1α	Hypoxia Pathway	[228]
CCAT2	Oncogene	↑	ERK	MAPK	[236]
PVT-1	Oncogene	↑	KLF-5,β-Catenin	WNT/β-Catenin	[228]
UCA1	Oncogene	↑	mTOR,β-Catenin	mTOR, WNT/ β-Catenin	[237,238]
GAS5	Tumor suppressor	↓	PTEN	Apoptosis	[239]
BCAR4	Oncogene	↑	SNIP1, PNUTS	Hedgehog /GLI 2 Signaling Transduction	[228]
NEAT	Oncogene	↑	ZEB1, RAS	RAS, MAPK, RSF1	[227]

## Abbreviations

ALDH1A3	Aldehyde Dehydrogenase 1A3
ANRIL	antisense to the CDKN2B locus
ASCO	American Society of Clinical Oncology
BC	Breast cancer
BCL-2	B-cell lymphoma 2
BCSC	Breast Cancer Stem Cells
CAF	cancer-associated fibroblast
CAFs	cancer-associated fibroblasts
CCAT2	Colon Cancer Associated Transcript 2
CCL2	monocyte chemotactic protein-1
CCL7	monocyte chemotactic protein-7
CCND1	cyclin D1
CDK6	Cyclin-Dependent Kinase 6
Cernan	Competing endogenous RNA
CRISPR	Clustered Regularly Interspaced Short Palindromic Repeats
CRNDE	colorectal neoplasia differentially expressed
CSC	Cancer stem cells
CTNNB1	b-catenin
E2F1	E2F transcription factor 1
E2F3	E2F transcription factor 3
E-BCSC	Epithelial
ECM	extracellular matrix
EIF4EBP1	Eukaryotic Translation Initiation Factor 4E Binding Protein 1
EMT	Epithelial-to-Mesenchymal Transition
ENCODE	Encyclopedia of DNA Elements project
ER	Estrogen receptor
ERBB2	Receptor tyrosine-protein kinase erbB-2
ErbB2/3	Receptor tyrosine-protein kinase 2/3
EZH2	Enhancer of Zester Homolog 2
FGF13-AS1	fibroblast growth factor 13-antisense RNA 1
FGF5	fibroblast growth factor 5
FOXC1	Forehead box C1
GAS5	growth arrest specific 5
HAS2	hyaluronic synthase
HIF 1α	Hypoxia-inducible factor-1
HOTAIR	HOX Transcript Antisense Intergenic RNA
HOX	Homeobox
KLF4	Rappel-Like Factor 4
lncRNA	Long intervening/intergenic noncoding RNAs
LINK-A	Long intergenic non-coding RNA for kinase activation
LncRNA	long non-coding RNA
LUCAT1	Lung Cancer Associated Transcript 1
MALAT1	metastasis-associated lung adenocarcinoma transcript 1
MAPK	Microtubule Associated Protein Kinase
Mass	mammary stem cells
M-BCSC	Mesenchymal
MEG3	Maternally Expressed Gene 3
MIAT	myocardial infarction associated transcript
miRNA	Micro RNA
mRNA	Messenger RNAs
MSCs	mesenchymal stem cells
MYC	MYC Proto-Oncogene
MYT-1	Myelin Transcription Factor 1
NCCN	National Comprehensive Cancer Network
NCID	Notch intracellular domain
ncRNAs	noncoding RNAs
NEAT1	nuclear Para speckle assembly transcript 1
NEDD4L	Neural precursor cell expressed developmentally down-regulated gene 4-like
NRAD1	non-coding RNA in the aldehyde dehydrogenase 1A pathway
OCT4	octamer-binding transcription factor 4
p53	protein p53
PARP	Poly ADP ribose polymerase
PDGF-BB	platelet derived growth factor BB
PDK1	Phosphoinositide-dependent kinase 1
PHLDA1	Pleckstrin homology-like domain, family A member 1
pine	Piwi-interacting RNA
PLAGL2	pleomorphic gene like-2
PR	Progesterone receptor
RMRP	RNA component of mitochondrial RNA processing endoribonuclease
RMST	Rhabdomyosarcoma 2-associated transcript
ROR	receptor tyrosine kinase-like orphan receptor
RUNX1	Chr. Runt-related transcription factor 1
S1P	sphingosine-1-phosphate
siRNA	small interfering Ribonucleic Acid
SIRT1	silent mating type information regulation 2 homolog
SnaR	steroid receptor RNA activator
sncRNAs	short noncoding RNAs
SNHG12	Small nucleolar RNA host gene 12
snoRNA	Small nucleolar RNAs
snRNA	Small nuclear RNA
SOX 2	Sry-related high mobility group box 2
Sox9	Sry-related HMG box 9
STAT3-CPTIB-FAO	JAK/STAT3-Regulated Fatty Acid β-Oxidation I
STXBP5-AS1	STXBP5 Antisense RNA 1
SUZ12	suppressor of zeste 12
TAM	Tumor-Associated Macrophages
TCF/LEF	T cell factor/lymphoid enhancing factor
TERC	Telomerase RNA component
TERRA	telomeric repeat-containing RNA
TET	ten-eleven translocation
TGF-β	Transforming growth factor beta
TME	tumor microenvironment
TNBC	Triple Negative Breast Cancer
Tregs	T regulatory cells
tRFs	tRNA-derived fragments
UCA1	urothelial carcinoma associated 1
XIST	X inactive specific transcript
ZBTB10	Zinc finger and BTB domain containing 10
ZEB 2	Zinc finger E-box binding homeobox 2
ZEB1	zinc finger E-box-binding homeobox 1

## References

[1] Bray F., Ferlay J., Soerjomataram I., Siegel R.L., Torre L.A., Jemal A. (2018). Global cancer statistics 2018: GLOBOCAN estimates of incidence and mortality worldwide for 36 cancers in 185 countries. CA Cancer J. Clin..

[2] Turashvili G., Brogi E. (2017). Tumor Heterogeneity in Breast Cancer. Front. Med. (Lausanne).

[3] Fragomeni S.M., Sciallis A., Jeruss J.S. (2018). Molecular Subtypes and Local-Regional Control of Breast Cancer. Surg. Oncol. Clin. N. Am..

[4] Perou C.M., Sorlie T., Eisen M.B., van de Rijn M., Jeffrey S.S., Rees C.A., Pollack J.R., Ross D.T., Johnsen H., Akslen L.A. (2000). Molecular portraits of human breast tumours. Nature.

[5] Sorlie T., Perou C.M., Tibshirani R., Aas T., Geisler S., Johnsen H., Hastie T., Eisen M.B., van de Rijn M., Jeffrey S.S. (2001). Gene expression patterns of breast carcinomas distinguish tumor subclasses with clinical implications. Proc. Natl. Acad. Sci. USA.

[6] Reis-Filho J.S., Pusztai L. (2011). Gene expression profiling in breast cancer: classification, prognostication, and prediction. Lancet.

[7] Gradishar W.J., Anderson B.O., Balassanian R., Blair S.L., Burstein H.J., Cyr A., Elias A.D., Farrar W.B., Forero A., Giordano S.H. (2017). NCCN Guidelines Insights: Breast Cancer, Version 1.2017. J. Natl. Compr. Canc. Netw..

[8] Breast Cancer (ASCO). https://ascopubs.org.doi/10.1200/EDBK_237715.

[9] Chan C.W.H., Law B.M.H., So W.K.W., Chow K.M., Waye M.M.Y. (2017). Novel Strategies on Personalized Medicine for Breast Cancer Treatment: An Update. Int. J. Mol. Sci..

[10] Djebali S., Davis C.A., Merkel A., Dobin A., Lassmann T., Mortazavi A., Tanzer A., Lagarde J., Lin W., Schlesinger F. (2012). Landscape of transcription in human cells. Nature.

[11] Iyer M.K., Niknafs Y.S., Malik R., Singhal U., Sahu A., Hosono Y., Barrette T.R., Prensner J.R., Evans J.R., Zhao S. (2015). The landscape of long noncoding RNAs in the human transcriptome. Nat. Genet..

[12] Prensner J.R., Iyer M.K., Balbin O.A., Dhanasekaran S.M., Cao Q., Brenner J.C., Laxman B., Asangani I.A., Grasso C.S., Kominsky H.D. (2011). Transcriptome sequencing across a prostate cancer cohort identifies PCAT-1, an unannotated lincRNA implicated in disease progression. Nat. Biotechnol..

[13] Pennisi E. (2012). Genomics. ENCODE project writes eulogy for junk DNA. Science.

[14] Romano G., Veneziano D., Acunzo M., Croce C.M. (2017). Small non-coding RNA and cancer. Carcinogenesis.

[15] O’Day E., Lal A. (2010). MicroRNAs and their target gene networks in breast cancer. Breast Cancer Res..

[16] Ma L., Bajic V.B., Zhang Z. (2013). On the classification of long non-coding RNAs. RNA Biol..

[17] Liu Y., Sharma S., Watabe K. (2015). Roles of lncRNA in breast cancer. Front. Biosci. (Schol. Ed.).

[18] Dykes I.M., Emanueli C. (2017). Transcriptional and Post-transcriptional Gene Regulation by Long Non-coding RNA. Genom. Proteom. Bioinf..

[19] Zhang J., Liu L., Li J., Le T.D. (2018). LncmiRSRN: identification and analysis of long non-coding RNA related miRNA sponge regulatory network in human cancer. Bioinformatics.

[20] Xue M., Zhuo Y., Shan B. (2017). MicroRNAs, Long Noncoding RNAs, and Their Functions in Human Disease. Methods Mol. Biol..

[21] Iorio M.V., Croce C.M. (2017). MicroRNA dysregulation in cancer: Diagnostics, monitoring and therapeutics. A comprehensive review. EMBO Mol. Med..

[22] He L., Hannon G.J. (2004). MicroRNAs: small RNAs with a big role in gene regulation. Nat. Rev. Genet..

[23] Hayes J., Peruzzi P.P., Lawler S. (2014). MicroRNAs in cancer: Biomarkers, functions and therapy. Trends Mol. Med..

[24] Derrien T., Johnson R., Bussotti G., Tanzer A., Djebali S., Tilgner H., Guernec G., Martin D., Merkel A., Knowles D.G. (2012). The GENCODE v7 catalog of human long noncoding RNAs: Analysis of their gene structure, evolution, and expression. Genome. Res..

[25] Wang H., Chung P.J., Liu J., Jang I.C., Kean M.J., Xu J., Chua N.H. (2014). Genome-wide identification of long noncoding natural antisense transcripts and their responses to light in Arabidopsis. Genome. Res..

[26] Bu D., Yu K., Sun S., Xie C., Skogerbo G., Miao R., Xiao H., Liao Q., Luo H., Zhao G. (2012). NONCODE v3.0: integrative annotation of long noncoding RNAs. Nucleic. Acids. Res..

[27] Cheng C., Sharp P.A. (2003). RNA polymerase II accumulation in the promoter-proximal region of the dihydrofolate reductase and gamma-actin genes. Mol. Cell. Biol..

[28] Geisler S., Coller J. (2013). RNA in unexpected places: Long non-coding RNA functions in diverse cellular contexts. Nat. Rev. Mol. Cell Biol..

[29] Hutchinson J.N., Ensminger A.W., Clemson C.M., Lynch C.R., Lawrence J.B., Chess A. (2007). A screen for nuclear transcripts identifies two linked noncoding RNAs associated with SC35 splicing domains. BMC Genomics.

[30] Kishore S., Gruber A.R., Jedlinski D.J., Syed A.P., Jorjani H., Zavolan M. (2013). Insights into snoRNA biogenesis and processing from PAR-CLIP of snoRNA core proteins and small RNA sequencing. Genome. Biol..

[31] Vicens Q., Westhof E. (2014). Biogenesis of Circular RNAs. Cell.

[32] Chen L.L., Yang L. (2015). Regulation of circRNA biogenesis. RNA Biol..

[33] Naganuma T., Hirose T. (2013). Paraspeckle formation during the biogenesis of long non-coding RNAs. RNA Biol..

[34] Fox A.H., Nakagawa S., Hirose T., Bond C.S. (2018). Paraspeckles: Where Long Noncoding RNA Meets Phase Separation. Trends Biochem. Sci..

[35] Tragante V., Moore J.H., Asselbergs F.W. (2014). The ENCODE project and perspectives on pathways. Genet. Epidemiol..

[36] Tang Q., Hann S.S. (2018). HOTAIR: An Oncogenic Long Non-Coding RNA in Human Cancer. Cell Physiol. Biochem..

[37] Vance K.W., Ponting C.P. (2014). Transcriptional regulatory functions of nuclear long noncoding RNAs. Trends Genet..

[38] DiStefano J.K. (2018). The Emerging Role of Long Noncoding RNAs in Human Disease. Methods Mol. Biol..

[39] Cipolla G.A., de Oliveira J.C., Salviano-Silva A., Lobo-Alves S.C., Lemos D.S., Oliveira L.C., Jucoski T.S., Mathias C., Pedroso G.A., Zambalde E.P. (2018). Long Non-Coding RNAs in Multifactorial Diseases: Another Layer of Complexity. Noncoding RNA.

[40] Zhang T., Hu H., Yan G., Wu T., Liu S., Chen W., Ning Y., Lu Z. (2019). Long Non-Coding RNA and Breast Cancer. Technol. Cancer Res. Treat..

[41] Gasch C., Ffrench B., O’Leary J.J., Gallagher M.F. (2017). Catching moving targets: cancer stem cell hierarchies, therapy-resistance & considerations for clinical intervention. Mol. Cancer.

[42] Yu Z., Pestell T.G., Lisanti M.P., Pestell R.G. (2012). Cancer stem cells. Int. J. Biochem. Cell Biol..

[43] Palomeras S., Ruiz-Martinez S., Puig T. (2018). Targeting Breast Cancer Stem Cells to Overcome Treatment Resistance. Molecules.

[44] Zhou J., Chen Q., Zou Y., Chen H., Qi L., Chen Y. (2019). Stem Cells and Cellular Origins of Breast Cancer: Updates in the Rationale, Controversies, and Therapeutic Implications. Front. Oncol..

[45] Liu S., Cong Y., Wang D., Sun Y., Deng L., Liu Y., Martin-Trevino R., Shang L., McDermott S.P., Landis M.D. (2014). Breast cancer stem cells transition between epithelial and mesenchymal states reflective of their normal counterparts. Stem. Cell Reports.

[46] Al-Hajj M., Wicha M.S., Benito-Hernandez A., Morrison S.J., Clarke M.F. (2003). Prospective identification of tumorigenic breast cancer cells. Proc. Natl Acad. Sci. USA.

[47] Prat A., Parker J.S., Karginova O., Fan C., Livasy C., Herschkowitz J.I., He X., Perou C.M. (2010). Phenotypic and molecular characterization of the claudin-low intrinsic subtype of breast cancer. Breast Cancer Res..

[48] LaBarge M.A., Petersen O.W., Bissell M.J. (2007). Of microenvironments and mammary stem cells. Stem Cell Rev..

[49] Wiseman B.S., Werb Z. (2002). Stromal effects on mammary gland development and breast cancer. Science.

[50] Silberstein G.B. (2001). Tumour-stromal interactions. Role of the stroma in mammary development. Breast Cancer Res..

[51] Parmar H., Cunha G.R. (2004). Epithelial-stromal interactions in the mouse and human mammary gland in vivo. Endocr. Relat. Cancer.

[52] Bocci F., Gearhart-Serna L., Boareto M., Ribeiro M., Ben-Jacob E., Devi G.R., Levine H., Onuchic J.N., Jolly M.K. (2019). Toward understanding cancer stem cell heterogeneity in the tumor microenvironment. Proc. Natl. Acad. Sci. USA.

[53] Bhat V., Allan A.L., Raouf A. (2019). Role of the Microenvironment in Regulating Normal and Cancer Stem Cell Activity: Implications for Breast Cancer Progression and Therapy Response. Cancers (Basel).

[54] Liubomirski Y., Lerrer S., Meshel T., Rubinstein-Achiasaf L., Morein D., Wiemann S., Korner C., Ben-Baruch A. (2019). Tumor-Stroma-Inflammation Networks Promote Pro-metastatic Chemokines and Aggressiveness Characteristics in Triple-Negative Breast Cancer. Front. Immunol..

[55] Korkaya H., Liu S., Wicha M.S. (2011). Breast cancer stem cells, cytokine networks, and the tumor microenvironment. J. Clin. Invest..

[56] Chatterjee S., Basak P., Buchel E., Safneck J., Murphy L.C., Mowat M., Kung S.K., Eirew P., Eaves C.J., Raouf A. (2018). Breast Cancers Activate Stromal Fibroblast-Induced Suppression of Progenitors in Adjacent Normal Tissue. Stem Cell Reports.

[57] Tsuyada A., Chow A., Wu J., Somlo G., Chu P., Loera S., Luu T., Li A.X., Wu X., Ye W. (2012). CCL2 mediates cross-talk between cancer cells and stromal fibroblasts that regulates breast cancer stem cells. Cancer Res..

[58] Ohlund D., Handly-Santana A., Biffi G., Elyada E., Almeida A.S., Ponz-Sarvise M., Corbo V., Oni T.E., Hearn S.A., Lee E.J. (2017). Distinct populations of inflammatory fibroblasts and myofibroblasts in pancreatic cancer. J. Exp. Med..

[59] Sugimoto H., Mundel T.M., Kieran M.W., Kalluri R. (2006). Identification of fibroblast heterogeneity in the tumor microenvironment. Cancer Biol. Ther..

[60] Cazet A.S., Hui M.N., Elsworth B.L., Wu S.Z., Roden D., Chan C.L., Skhinas J.N., Collot R., Yang J., Harvey K. (2018). Targeting stromal remodeling and cancer stem cell plasticity overcomes chemoresistance in triple negative breast cancer. Nat. Commun..

[61] Valenti G., Quinn H.M., Heynen G., Lan L., Holland J.D., Vogel R., Wulf-Goldenberg A., Birchmeier W. (2017). Cancer Stem Cells Regulate Cancer-Associated Fibroblasts via Activation of Hedgehog Signaling in Mammary Gland Tumors. Cancer Res..

[62] Al-Khalaf H.H., Ghebeh H., Inass R., Aboussekhra A. (2019). Senescent Breast Luminal Cells Promote Carcinogenesis through Interleukin-8-Dependent Activation of Stromal Fibroblasts. Mol. Cell Biol..

[63] Su S., Chen J., Yao H., Liu J., Yu S., Lao L., Wang M., Luo M., Xing Y., Chen F. (2018). CD10(+)GPR77(+) Cancer-Associated Fibroblasts Promote Cancer Formation and Chemoresistance by Sustaining Cancer Stemness. Cell.

[64] Liu S., Ginestier C., Ou S.J., Clouthier S.G., Patel S.H., Monville F., Korkaya H., Heath A., Dutcher J., Kleer C.G. (2011). Breast cancer stem cells are regulated by mesenchymal stem cells through cytokine networks. Cancer Res..

[65] Ma X.J., Dahiya S., Richardson E., Erlander M., Sgroi D.C. (2009). Gene expression profiling of the tumor microenvironment during breast cancer progression. Breast Cancer Res..

[66] Yang J., Liao D., Chen C., Liu Y., Chuang T.H., Xiang R., Markowitz D., Reisfeld R.A., Luo Y. (2013). Tumor-associated macrophages regulate murine breast cancer stem cells through a novel paracrine EGFR/Stat3/Sox-2 signaling pathway. Stem Cells.

[67] Okuda H., Kobayashi A., Xia B., Watabe M., Pai S.K., Hirota S., Xing F., Liu W., Pandey P.R., Fukuda K. (2012). Hyaluronan synthase HAS2 promotes tumor progression in bone by stimulating the interaction of breast cancer stem-like cells with macrophages and stromal cells. Cancer Res..

[68] Lu H., Clauser K.R., Tam W.L., Frose J., Ye X., Eaton E.N., Reinhardt F., Donnenberg V.S., Bhargava R., Carr S.A. (2014). A breast cancer stem cell niche supported by juxtacrine signalling from monocytes and macrophages. Nat. Cell Biol..

[69] Nalla L.V., Kalia K., Khairnar A. (2019). Self-renewal signaling pathways in breast cancer stem cells. Int J Biochem. Cell Biol..

[70] Al-Hussaini H., Subramanyam D., Reedijk M., Sridhar S.S. (2011). Notch signaling pathway as a therapeutic target in breast cancer. Mol. Cancer Ther..

[71] Habib J.G., O’Shaughnessy J.A. (2016). The hedgehog pathway in triple-negative breast cancer. Cancer Med..

[72] King T.D., Suto M.J., Li Y. (2012). The Wnt/beta-catenin signaling pathway: A potential therapeutic target in the treatment of triple negative breast cancer. J. Cell Biochem..

[73] Borah A., Raveendran S., Rochani A., Maekawa T., Kumar D.S. (2015). Targeting self-renewal pathways in cancer stem cells: clinical implications for cancer therapy. Oncogenesis.

[74] Matsui W.H. (2016). Cancer stem cell signaling pathways. Medicine (Baltimore).

[75] Johnson D.E., O’Keefe R.A., Grandis J.R. (2018). Targeting the IL-6/JAK/STAT3 signalling axis in cancer. Nat. Rev. Clin. Oncol..

[76] Peng D., Tanikawa T., Li W., Zhao L., Vatan L., Szeliga W., Wan S., Wei S., Wang Y., Liu Y. (2016). Myeloid-Derived Suppressor Cells Endow Stem-like Qualities to Breast Cancer Cells through IL6/STAT3 and NO/NOTCH Cross-talk Signaling. Cancer Res..

[77] Fang X., Cai Y., Liu J., Wang Z., Wu Q., Zhang Z., Yang C.J., Yuan L., Ouyang G. (2011). Twist2 contributes to breast cancer progression by promoting an epithelial-mesenchymal transition and cancer stem-like cell self-renewal. Oncogene.

[78] Thiagarajan P.S., Zheng Q., Bhagrath M., Mulkearns-Hubert E.E., Myers M.G., Lathia J.D., Reizes O. (2017). STAT3 activation by leptin receptor is essential for TNBC stem cell maintenance. Endocr. Relat. Cancer.

[79] Wang T., Fahrmann J.F., Lee H., Li Y.J., Tripathi S.C., Yue C., Zhang C., Lifshitz V., Song J., Yuan Y. (2018). JAK/STAT3-Regulated Fatty Acid beta-Oxidation Is Critical for Breast Cancer Stem Cell Self-Renewal and Chemoresistance. Cell Metab..

[80] Zhong Y., Shen S., Zhou Y., Mao F., Lin Y., Guan J., Xu Y., Zhang S., Liu X., Sun Q. (2016). NOTCH1 is a poor prognostic factor for breast cancer and is associated with breast cancer stem cells. Onco. Targets Ther..

[81] Gonzalez M.E., Moore H.M., Li X., Toy K.A., Huang W., Sabel M.S., Kidwell K.M., Kleer C.G. (2014). EZH2 expands breast stem cells through activation of NOTCH1 signaling. Proc. Natl. Acad. Sci. USA.

[82] Hirata N., Yamada S., Shoda T., Kurihara M., Sekino Y., Kanda Y. (2014). Sphingosine-1-phosphate promotes expansion of cancer stem cells via S1PR3 by a ligand-independent Notch activation. Nat. Commun..

[83] Mohammed M.K., Shao C., Wang J., Wei Q., Wang X., Collier Z., Tang S., Liu H., Zhang F., Huang J. (2016). Wnt/beta-catenin signaling plays an ever-expanding role in stem cell self-renewal, tumorigenesis and cancer chemoresistance. Genes Dis..

[84] Domenici G., Aurrekoetxea-Rodriguez I., Simoes B.M., Rabano M., Lee S.Y., Millan J.S., Comaills V., Oliemuller E., Lopez-Ruiz J.A., Zabalza I. (2019). A Sox2-Sox9 signalling axis maintains human breast luminal progenitor and breast cancer stem cells. Oncogene.

[85] Protecting Workers’ Health. https://www.who.int/news-room/fact-sheets/detail/protecting-workers’-health.

[86] Wang L., Duan W., Kang L., Mao J., Yu X., Fan S., Li L., Tao Y. (2014). Smoothened activates breast cancer stem-like cell and promotes tumorigenesis and metastasis of breast cancer. Biomed. Pharmacother..

[87] Han B., Qu Y., Jin Y., Yu Y., Deng N., Wawrowsky K., Zhang X., Li N., Bose S., Wang Q. (2015). FOXC1 Activates Smoothened-Independent Hedgehog Signaling in Basal-like Breast Cancer. Cell Rep..

[88] Loh H.Y., Norman B.P., Lai K.S., Rahman N., Alitheen N.B.M., Osman M.A. (2019). The Regulatory Role of MicroRNAs in Breast Cancer. Int. J. Mol. Sci..

[89] Luo Q., Li X., Gao Y., Long Y., Chen L., Huang Y., Fang L. (2013). MiRNA-497 regulates cell growth and invasion by targeting cyclin E1 in breast cancer. Cancer Cell Int..

[90] Guo X., Connick M.C., Vanderhoof J., Ishak M.A., Hartley R.S. (2015). MicroRNA-16 modulates HuR regulation of cyclin E1 in breast cancer cells. Int. J. Mol. Sci..

[91] Shukla K., Sharma A.K., Ward A., Will R., Hielscher T., Balwierz A., Breunig C., Munstermann E., Konig R., Keklikoglou I. (2015). MicroRNA-30c-2-3p negatively regulates NF-kappaB signaling and cell cycle progression through downregulation of TRADD and CCNE1 in breast cancer. Mol. Oncol..

[92] Huang X., Lyu J. (2018). Tumor suppressor function of miR-483-3p on breast cancer via targeting of the cyclin E1 gene. Exp. Ther. Med..

[93] Yan C., Chen Y., Kong W., Fu L., Liu Y., Yao Q., Yuan Y. (2017). PVT1-derived miR-1207-5p promotes breast cancer cell growth by targeting STAT6. Cancer Sci..

[94] Jiang Q., He M., Ma M.T., Wu H.Z., Yu Z.J., Guan S., Jiang L.Y., Wang Y., Zheng D.D., Jin F. (2016). MicroRNA-148a inhibits breast cancer migration and invasion by directly targeting WNT-1. Oncol. Rep..

[95] Mohammadi-Yeganeh S., Paryan M., Arefian E., Vasei M., Ghanbarian H., Mahdian R., Karimipoor M., Soleimani M. (2016). MicroRNA-340 inhibits the migration, invasion, and metastasis of breast cancer cells by targeting Wnt pathway. Tumour Biol.

[96] Yokota T., Furukawa T., Tsukagoshi H. (1989). Motor paresis improved by sympathetic block. A motor form of reflex sympathetic dystrophy?. Arch Neurol.

[97] Pan Y., Jiao G., Wang C., Yang J., Yang W. (2016). MicroRNA-421 inhibits breast cancer metastasis by targeting metastasis associated 1. Biomed. Pharmacother..

[98] Xie F., Hosany S., Zhong S., Jiang Y., Zhang F., Lin L., Wang X., Gao S., Hu X. (2017). MicroRNA-193a inhibits breast cancer proliferation and metastasis by downregulating WT1. PLoS ONE.

[99] Liu C., Liu Z., Li X., Tang X., He J., Lu S. (2017). MicroRNA-1297 contributes to tumor growth of human breast cancer by targeting PTEN/PI3K/AKT signaling. Oncol. Rep..

[100] Miao Y., Zheng W., Li N., Su Z., Zhao L., Zhou H., Jia L. (2017). MicroRNA-130b targets PTEN to mediate drug resistance and proliferation of breast cancer cells via the PI3K/Akt signaling pathway. Sci. Rep..

[101] Hong B.S., Ryu H.S., Kim N., Kim J., Lee E., Moon H., Kim K.H., Jin M.S., Kwon N.H., Kim S. (2019). Tumor Suppressor miRNA-204-5p Regulates Growth, Metastasis, and Immune Microenvironment Remodeling in Breast Cancer. Cancer Res..

[102] Khan A.Q., Ahmed E.I., Elareer N.R., Junejo K., Steinhoff M., Uddin S. (2019). Role of miRNA-Regulated Cancer Stem Cells in the Pathogenesis of Human Malignancies. Cells.

[103] Fan X., Chen W., Fu Z., Zeng L., Yin Y., Yuan H. (2017). MicroRNAs, a subpopulation of regulators, are involved in breast cancer progression through regulating breast cancer stem cells. Oncol. Lett..

[104] Lim Y.Y., Wright J.A., Attema J.L., Gregory P.A., Bert A.G., Smith E., Thomas D., Lopez A.F., Drew P.A., Khew-Goodall Y. (2013). Epigenetic modulation of the miR-200 family is associated with transition to a breast cancer stem-cell-like state. J. Cell Sci..

[105] Polytarchou C., Iliopoulos D., Struhl K. (2012). An integrated transcriptional regulatory circuit that reinforces the breast cancer stem cell state. Proc. Natl. Acad. Sci. USA.

[106] Iliopoulos D., Lindahl-Allen M., Polytarchou C., Hirsch H.A., Tsichlis P.N., Struhl K. (2010). Loss of miR-200 inhibition of Suz12 leads to polycomb-mediated repression required for the formation and maintenance of cancer stem cells. Mol. Cell.

[107] Wellner U., Schubert J., Burk U.C., Schmalhofer O., Zhu F., Sonntag A., Waldvogel B., Vannier C., Darling D., zur Hausen A. (2009). The EMT-activator ZEB1 promotes tumorigenicity by repressing stemness-inhibiting microRNAs. Nat. Cell. Biol..

[108] Dykxhoorn D.M., Wu Y., Xie H., Yu F., Lal A., Petrocca F., Martinvalet D., Song E., Lim B., Lieberman J. (2009). miR-200 enhances mouse breast cancer cell colonization to form distant metastases. PLoS ONE.

[109] Knezevic J., Pfefferle A.D., Petrovic I., Greene S.B., Perou C.M., Rosen J.M. (2015). Expression of miR-200c in claudin-low breast cancer alters stem cell functionality, enhances chemosensitivity and reduces metastatic potential. Oncogene.

[110] van den Beucken T., Koch E., Chu K., Rupaimoole R., Prickaerts P., Adriaens M., Voncken J.W., Harris A.L., Buffa F.M., Haider S. (2014). Hypoxia promotes stem cell phenotypes and poor prognosis through epigenetic regulation of DICER. Nat. Commun..

[111] Song S.J., Poliseno L., Song M.S., Ala U., Webster K., Ng C., Beringer G., Brikbak N.J., Yuan X., Cantley L.C. (2013). MicroRNA-antagonism regulates breast cancer stemness and metastasis via TET-family dependent chromatin remodeling. Cell.

[112] Valastyan S., Chang A., Benaich N., Reinhardt F., Weinberg R.A. (2010). Concurrent suppression of integrin alpha5, radixin, and RhoA phenocopies the effects of miR-31 on metastasis. Cancer Res..

[113] Valastyan S., Reinhardt F., Benaich N., Calogrias D., Szasz A.M., Wang Z.C., Brock J.E., Richardson A.L., Weinberg R.A. (2009). A pleiotropically acting microRNA, miR-31, inhibits breast cancer metastasis. Cell.

[114] Sachdeva M., Mo Y.Y. (2010). MicroRNA-145 suppresses cell invasion and metastasis by directly targeting mucin 1. Cancer Res..

[115] Spizzo R., Nicoloso M.S., Lupini L., Lu Y., Fogarty J., Rossi S., Zagatti B., Fabbri M., Veronese A., Liu X. (2010). miR-145 participates with TP53 in a death-promoting regulatory loop and targets estrogen receptor-alpha in human breast cancer cells. Cell Death. Differ..

[116] Wang S., Bian C., Yang Z., Bo Y., Li J., Zeng L., Zhou H., Zhao R.C. (2009). miR-145 inhibits breast cancer cell growth through RTKN. Int. J. Oncol..

[117] Jiang S., Zhang H.W., Lu M.H., He X.H., Li Y., Gu H., Liu M.F., Wang E.D. (2010). MicroRNA-155 functions as an OncomiR in breast cancer by targeting the suppressor of cytokine signaling 1 gene. Cancer Res..

[118] Kong W., He L., Coppola M., Guo J., Esposito N.N., Coppola D., Cheng J.Q. (2010). MicroRNA-155 regulates cell survival, growth, and chemosensitivity by targeting FOXO3a in breast cancer. J. Biol. Chem..

[119] Kong W., Yang H., He L., Zhao J.J., Coppola D., Dalton W.S., Cheng J.Q. (2008). MicroRNA-155 is regulated by the transforming growth factor beta/Smad pathway and contributes to epithelial cell plasticity by targeting RhoA. Mol. Cell Biol..

[120] Carpenter R.L., Paw I., Dewhirst M.W., Lo H.W. (2015). Akt phosphorylates and activates HSF-1 independent of heat shock, leading to Slug overexpression and epithelial-mesenchymal transition (EMT) of HER2-overexpressing breast cancer cells. Oncogene.

[121] Song B., Wang C., Liu J., Wang X., Lv L., Wei L., Xie L., Zheng Y., Song X. (2010). MicroRNA-21 regulates breast cancer invasion partly by targeting tissue inhibitor of metalloproteinase 3 expression. J. Exp. Clin. Cancer Res..

[122] Qi L., Bart J., Tan L.P., Platteel I., Sluis T., Huitema S., Harms G., Fu L., Hollema H., Berg A. (2009). Expression of miR-21 and its targets (PTEN, PDCD4, TM1) in flat epithelial atypia of the breast in relation to ductal carcinoma in situ and invasive carcinoma. BMC Cancer.

[123] Huang G.L., Zhang X.H., Guo G.L., Huang K.T., Yang K.Y., Hu X.Q. (2008). Expression of microRNA-21 in invasive ductal carcinoma of the breast and its association with phosphatase and tensin homolog deleted from chromosome expression and clinicopathologic features. Chinese Med. J..

[124] Qian B., Katsaros D., Lu L., Preti M., Durando A., Arisio R., Mu L., Yu H. (2009). High miR-21 expression in breast cancer associated with poor disease-free survival in early stage disease and high TGF-beta1. Breast Cancer Res. Treat..

[125] Scott G.K., Goga A., Bhaumik D., Berger C.E., Sullivan C.S., Benz C.C. (2007). Coordinate suppression of ERBB2 and ERBB3 by enforced expression of micro-RNA miR-125a or miR-125b. J. Biol. Chem..

[126] Zhou M., Liu Z., Zhao Y., Ding Y., Liu H., Xi Y., Xiong W., Li G., Lu J., Fodstad O. (2010). MicroRNA-125b confers the resistance of breast cancer cells to paclitaxel through suppression of pro-apoptotic Bcl-2 antagonist killer 1 (Bak1) expression. J. Biol. Chem..

[127] Hofmann M.H., Heinrich J., Radziwill G., Moelling K. (2009). A short hairpin DNA analogous to miR-125b inhibits C-Raf expression, proliferation, and survival of breast cancer cells. Mol. Cancer Res..

[128] Ma L., Reinhardt F., Pan E., Soutschek J., Bhat B., Marcusson E.G., Teruya-Feldstein J., Bell G.W., Weinberg R.A. (2010). Therapeutic silencing of miR-10b inhibits metastasis in a mouse mammary tumor model. Nat. Biotechnol..

[129] Ma L., Teruya-Feldstein J., Weinberg R.A. (2007). Tumour invasion and metastasis initiated by microRNA-10b in breast cancer. Nature.

[130] Ahmad A., Ginnebaugh K.R., Yin S., Bollig-Fischer A., Reddy K.B., Sarkar F.H. (2015). Functional role of miR-10b in tamoxifen resistance of ER-positive breast cancer cells through down-regulation of HDAC4. BMC Cancer.

[131] Iorio M.V., Ferracin M., Liu C.G., Veronese A., Spizzo R., Sabbioni S., Magri E., Pedriali M., Fabbri M., Campiglio M. (2005). MicroRNA gene expression deregulation in human breast cancer. Cancer Res..

[132] Wu H., Zhu S., Mo Y.Y. (2009). Suppression of cell growth and invasion by miR-205 in breast cancer. Cell Res..

[133] Gregory P.A., Bert A.G., Paterson E.L., Barry S.C., Tsykin A., Farshid G., Vadas M.A., Khew-Goodall Y., Goodall G.J. (2008). The miR-200 family and miR-205 regulate epithelial-to-mesenchymal transition by targeting ZEB1 and SIP1. Nat. Cell Biol..

[134] Camps C., Buffa F.M., Colella S., Moore J., Sotiriou C., Sheldon H., Harris A.L., Gleadle J.M., Ragoussis J. (2008). hsa-miR-210 Is induced by hypoxia and is an independent prognostic factor in breast cancer. Clin. Cancer Res..

[135] Zhang Z., Sun H., Dai H., Walsh R.M., Imakura M., Schelter J., Burchard J., Dai X., Chang A.N., Diaz R.L. (2009). MicroRNA miR-210 modulates cellular response to hypoxia through the MYC antagonist MNT. Cell Cycle.

[136] Luthra R., Singh R.R., Luthra M.G., Li Y.X., Hannah C., Romans A.M., Barkoh B.A., Chen S.S., Ensor J., Maru D.M. (2008). MicroRNA-196a targets annexin A1: a microRNA-mediated mechanism of annexin A1 downregulation in cancers. Oncogene.

[137] He H., Tian W., Chen H., Jiang K. (2016). MiR-944 functions as a novel oncogene and regulates the chemoresistance in breast cancer. Tumour. Biol..

[138] Shen H., Wang D., Li L., Yang S., Chen X., Zhou S., Zhong S., Zhao J., Tang J. (2017). MiR-222 promotes drug-resistance of breast cancer cells to adriamycin via modulation of PTEN/Akt/FOXO1 pathway. Gene.

[139] Zhang X., Zhong S., Xu Y., Yu D., Ma T., Chen L., Zhao Y., Chen X., Yang S., Wu Y. (2016). MicroRNA-3646 Contributes to Docetaxel Resistance in Human Breast Cancer Cells by GSK-3beta/beta-Catenin Signaling Pathway. PLoS ONE.

[140] Kastl L., Brown I., Schofield A.C. (2012). miRNA-34a is associated with docetaxel resistance in human breast cancer cells. Breast Cancer Res. Treat..

[141] Yao Y.S., Qiu W.S., Yao R.Y., Zhang Q., Zhuang L.K., Zhou F., Sun L.B., Yue L. (2015). miR-141 confers docetaxel chemoresistance of breast cancer cells via regulation of EIF4E expression. Oncol. Rep..

[142] Su C.M., Wang M.Y., Hong C.C., Chen H.A., Su Y.H., Wu C.H., Huang M.T., Chang Y.W., Jiang S.S., Sung S.Y. (2016). miR-520h is crucial for DAPK2 regulation and breast cancer progression. Oncogene.

[143] Kato M., Paranjape T., Muller R.U., Nallur S., Gillespie E., Keane K., Esquela-Kerscher A., Weidhaas J.B., Slack F.J. (2009). The mir-34 microRNA is required for the DNA damage response in vivo in C. elegans and in vitro in human breast cancer cells. Oncogene.

[144] Bhaumik D., Scott G.K., Schokrpur S., Patil C.K., Campisi J., Benz C.C. (2008). Expression of microRNA-146 suppresses NF-kappaB activity with reduction of metastatic potential in breast cancer cells. Oncogene.

[145] Reddy S.D., Ohshiro K., Rayala S.K., Kumar R. (2008). MicroRNA-7, a homeobox D10 target, inhibits p21-activated kinase 1 and regulates its functions. Cancer Res.

[146] Pandey D.P., Picard D. (2009). miR-22 inhibits estrogen signaling by directly targeting the estrogen receptor alpha mRNA. Mol Cell Biol.

[147] Rao X., Di Leva G., Li M., Fang F., Devlin C., Hartman-Frey C., Burow M.E., Ivan M., Croce C.M., Nephew K.P. (2011). MicroRNA-221/222 confers breast cancer fulvestrant resistance by regulating multiple signaling pathways. Oncogene.

[148] Nagpal N., Ahmad H.M., Molparia B., Kulshreshtha R. (2013). MicroRNA-191, an estrogen-responsive microRNA, functions as an oncogenic regulator in human breast cancer. Carcinogenesis.

[149] Tavazoie S.F., Alarcon C., Oskarsson T., Padua D., Wang Q., Bos P.D., Gerald W.L., Massague J. (2008). Endogenous human microRNAs that suppress breast cancer metastasis. Nature.

[150] Trompeter H.I., Abbad H., Iwaniuk K.M., Hafner M., Renwick N., Tuschl T., Schira J., Muller H.W., Wernet P. (2011). MicroRNAs MiR-17, MiR-20a, and MiR-106b act in concert to modulate E2F activity on cell cycle arrest during neuronal lineage differentiation of USSC. PLoS ONE.

[151] Ma L., Young J., Prabhala H., Pan E., Mestdagh P., Muth D., Teruya-Feldstein J., Reinhardt F., Onder T.T., Valastyan S. (2010). miR-9, a MYC/MYCN-activated microRNA, regulates E-cadherin and cancer metastasis. Nat. Cell Biol..

[152] Xia P., Wang Z., Liu X., Wu B., Wang J., Ward T., Zhang L., Ding X., Gibbons G., Shi Y. (2012). EB1 acetylation by P300/CBP-associated factor (PCAF) ensures accurate kinetochore-microtubule interactions in mitosis. Proc. Natl. Acad. Sci. USA.

[153] Zhu N., Zhang D., Xie H., Zhou Z., Chen H., Hu T., Bai Y., Shen Y., Yuan W., Jing Q. (2011). Endothelial-specific intron-derived miR-126 is down-regulated in human breast cancer and targets both VEGFA and PIK3R2. Mol. Cell Biochem..

[154] Siragam V., Rutnam Z.J., Yang W., Fang L., Luo L., Yang X., Li M., Deng Z., Qian J., Peng C. (2012). MicroRNA miR-98 inhibits tumor angiogenesis and invasion by targeting activin receptor-like kinase-4 and matrix metalloproteinase-11. Oncotarget.

[155] Xu Q., Jiang Y., Yin Y., Li Q., He J., Jing Y., Qi Y.T., Xu Q., Li W., Lu B. (2013). A regulatory circuit of miR-148a/152 and DNMT1 in modulating cell transformation and tumor angiogenesis through IGF-IR and IRS1. J. Mol. Cell Biol..

[156] Cha S.T., Chen P.S., Johansson G., Chu C.Y., Wang M.Y., Jeng Y.M., Yu S.L., Chen J.S., Chang K.J., Jee S.H. (2010). MicroRNA-519c suppresses hypoxia-inducible factor-1alpha expression and tumor angiogenesis. Cancer Res..

[157] Plummer P.N., Freeman R., Taft R.J., Vider J., Sax M., Umer B.A., Gao D., Johns C., Mattick J.S., Wilton S.D. (2013). MicroRNAs regulate tumor angiogenesis modulated by endothelial progenitor cells. Cancer Res..

[158] Lu Y., Qin T., Li J., Wang L., Zhang Q., Jiang Z., Mao J. (2017). MicroRNA-140-5p inhibits invasion and angiogenesis through targeting VEGF-A in breast cancer. Cancer Gene Ther..

[159] Liu Y., Lai L., Chen Q., Song Y., Xu S., Ma F., Wang X., Wang J., Yu H., Cao X. (2012). MicroRNA-494 is required for the accumulation and functions of tumor-expanded myeloid-derived suppressor cells via targeting of PTEN. J. Immunol..

[160] Liang Z., Bian X., Shim H. (2016). Downregulation of microRNA-206 promotes invasion and angiogenesis of triple negative breast cancer. Biochem. Biophys. Res. Commun..

[161] Anfossi S., Giordano A., Gao H., Cohen E.N., Tin S., Wu Q., Garza R.J., Debeb B.G., Alvarez R.H., Valero V. (2014). High serum miR-19a levels are associated with inflammatory breast cancer and are predictive of favorable clinical outcome in patients with metastatic HER2+ inflammatory breast cancer. PLoS ONE.

[162] Taguchi A., Yanagisawa K., Tanaka M., Cao K., Matsuyama Y., Goto H., Takahashi T. (2008). Identification of hypoxia-inducible factor-1 alpha as a novel target for miR-17-92 microRNA cluster. Cancer Res..

[163] Bhattacharyya S., Sul K., Krukovets I., Nestor C., Li J., Adognravi O.S. (2012). Novel tissue-specific mechanism of regulation of angiogenesis and cancer growth in response to hyperglycemia. J. Am. Heart Assoc..

[164] Bishnoi V., Kumar B., Bhagat H., Salunke P., Bishnoi S. (2016). Comparison of Dexmedetomidine Versus Midazolam-Fentanyl Combination for Monitored Anesthesia Care During Burr-Hole Surgery for Chronic Subdural Hematoma. J. Neurosurg. Anesthesiol..

[165] Tomar D., Yadav A.S., Kumar D., Bhadauriya G., Kundu G.C. (2019). Non-coding RNAs as potential therapeutic targets in breast cancer. Biochim. Biophys. Acta. Gene. Regul. Mech..

[166] Du Y.E., Tu G., Yang G., Li G., Yang D., Lang L., Xi L., Sun K., Chen Y., Shu K. (2017). MiR-205/YAP1 in Activated Fibroblasts of Breast Tumor Promotes VEGF-independent Angiogenesis through STAT3 Signaling. Theranostics.

[167] Jiang L., Yu L., Zhang X., Lei F., Wang L., Liu X., Wu S., Zhu J., Wu G., Cao L. (2016). miR-892b Silencing Activates NF-kappaB and Promotes Aggressiveness in Breast Cancer. Cancer Res..

[168] Jung E.J., Santarpia L., Kim J., Esteva F.J., Moretti E., Buzdar A.U., Di Leo A., Le X.F., Bast R.C., Park S.T. (2012). Plasma microRNA 210 levels correlate with sensitivity to trastuzumab and tumor presence in breast cancer patients. Cancer.

[169] Rothe F., Ignatiadis M., Chaboteaux C., Haibe-Kains B., Kheddoumi N., Majjaj S., Badran B., Fayyad-Kazan H., Desmedt C., Harris A.L. (2011). Global microRNA expression profiling identifies MiR-210 associated with tumor proliferation, invasion and poor clinical outcome in breast cancer. PLoS ONE.

[170] Gu X., Li J.Y., Guo J., Li P.S., Zhang W.H. (2015). Influence of MiR-451 on Drug Resistances of Paclitaxel-Resistant Breast Cancer Cell Line. Med. Sci. Monit..

[171] Zhang B., Zhao R., He Y., Fu X., Fu L., Zhu Z., Fu L., Dong J.T. (2016). MicroRNA 100 sensitizes luminal A breast cancer cells to paclitaxel treatment in part by targeting mTOR. Oncotarget.

[172] Zhang H.D., Sun D.W., Mao L., Zhang J., Jiang L.H., Li J., Wu Y., Ji H., Chen W., Wang J. (2015). MiR-139-5p inhibits the biological function of breast cancer cells by targeting Notch1 and mediates chemosensitivity to docetaxel. Biochem. Biophys. Res. Commun..

[173] Yu X., Luo A., Liu Y., Wang S., Li Y., Shi W., Liu Z., Qu X. (2015). MiR-214 increases the sensitivity of breast cancer cells to tamoxifen and fulvestrant through inhibition of autophagy. Mol. Cancer.

[174] Esteva F.J., Yu D., Hung M.C., Hortobagyi G.N. (2010). Molecular predictors of response to trastuzumab and lapatinib in breast cancer. Nat. Rev. Clin. Oncol..

[175] Fan X., Zhou S., Zheng M., Deng X., Yi Y., Huang T. (2017). MiR-199a-3p enhances breast cancer cell sensitivity to cisplatin by downregulating TFAM (TFAM). Biomed. Pharmacother..

[176] Cataldo A., Cheung D.G., Balsari A., Tagliabue E., Coppola V., Iorio M.V., Palmieri D., Croce C.M. (2016). miR-302b enhances breast cancer cell sensitivity to cisplatin by regulating E2F1 and the cellular DNA damage response. Oncotarget.

[177] He X., Xiao X., Dong L., Wan N., Zhou Z., Deng H., Zhang X. (2015). MiR-218 regulates cisplatin chemosensitivity in breast cancer by targeting BRCA1. Tumour. Biol..

[178] Tan X., Peng J., Fu Y., An S., Rezaei K., Tabbara S., Teal C.B., Man Y.G., Brem R.F., Fu S.W. (2014). miR-638 mediated regulation of BRCA1 affects DNA repair and sensitivity to UV and cisplatin in triple-negative breast cancer. Breast Cancer Res..

[179] Zhong S., Li W., Chen Z., Xu J., Zhao J. (2013). MiR-222 and miR-29a contribute to the drug-resistance of breast cancer cells. Gene.

[180] Zhang Y., Wang Y., Wei Y., Li M., Yu S., Ye M., Zhang H., Chen S., Liu W., Zhang J. (2015). MiR-129-3p promotes docetaxel resistance of breast cancer cells via CP110 inhibition. Sci. Rep..

[181] Zhang X., Yu H., Lou J.R., Zheng J., Zhu H., Popescu N.I., Lupu F., Lind S.E., Ding W.Q. (2011). MicroRNA-19 (miR-19) regulates tissue factor expression in breast cancer cells. J. Biol. Chem..

[182] He L., He X., Lim L.P., de Stanchina E., Xuan Z., Liang Y., Xue W., Zender L., Magnus J., Ridzon D. (2007). A microRNA component of the p53 tumour suppressor network. Nature.

[183] Yu F., Jiao Y., Zhu Y., Wang Y., Zhu J., Cui X., Liu Y., He Y., Park E.Y., Zhang H. (2012). MicroRNA 34c gene down-regulation via DNA methylation promotes self-renewal and epithelial-mesenchymal transition in breast tumor-initiating cells. J Biol Chem.

[184] Lin X., Chen W., Wei F., Zhou B.P., Hung M.C., Xie X. (2017). Nanoparticle Delivery of miR-34a Eradicates Long-term-cultured Breast Cancer Stem Cells via Targeting C22ORF28 Directly. Theranostics.

[185] Christoffersen N.R., Shalgi R., Frankel L.B., Leucci E., Lees M., Klausen M., Pilpel Y., Nielsen F.C., Oren M., Lund A.H. (2010). p53-independent upregulation of miR-34a during oncogene-induced senescence represses MYC. Cell Death. Differ..

[186] Welch C., Chen Y., Stallings R.L. (2007). MicroRNA-34a functions as a potential tumor suppressor by inducing apoptosis in neuroblastoma cells. Oncogene.

[187] Yamakuchi M., Ferlito M., Lowenstein C.J. (2008). miR-34a repression of SIRT1 regulates apoptosis. Proc. Natl. Acad. Sci. USA.

[188] Kang L., Mao J., Tao Y., Song B., Ma W., Lu Y., Zhao L., Li J., Yang B., Li L. (2015). MicroRNA-34a suppresses the breast cancer stem cell-like characteristics by downregulating Notch1 pathway. Cancer Sci..

[189] Guarnieri A.L., Towers C.G., Drasin D.J., Oliphant M.U.J., Andrysik Z., Hotz T.J., Vartuli R.L., Linklater E.S., Pandey A., Khanal S. (2018). The miR-106b-25 cluster mediates breast tumor initiation through activation of NOTCH1 via direct repression of NEDD4L. Oncogene.

[190] Wang H.J., Guo Y.Q., Tan G., Dong L., Cheng L., Li K.J., Wang Z.Y., Luo H.F. (2013). miR-125b regulates side population in breast cancer and confers a chemoresistant phenotype. J. Cell. Biochem..

[191] Wang Y., Yu Y., Tsuyada A., Ren X., Wu X., Stubblefield K., Rankin-Gee E.K., Wang S.E. (2011). Transforming growth factor-beta regulates the sphere-initiating stem cell-like feature in breast cancer through miRNA-181 and ATM. Oncogene.

[192] Niu J., Xue A., Chi Y., Xue J., Wang W., Zhao Z., Fan M., Yang C.H., Shao Z.M., Pfeffer L.M. (2016). Induction of miRNA-181a by genotoxic treatments promotes chemotherapeutic resistance and metastasis in breast cancer. Oncogene.

[193] Kastrati I., Canestrari E., Frasor J. (2015). PHLDA1 expression is controlled by an estrogen receptor-NFkappaB-miR-181 regulatory loop and is essential for formation of ER+ mammospheres. Oncogene.

[194] Min S., Li L., Zhang M., Zhang Y., Liang X., Xie Y., He Q., Li Y., Sun J., Liu Q. (2012). TGF-beta-associated miR-27a inhibits dendritic cell-mediated differentiation of Th1 and Th17 cells by TAB3, p38 MAPK, MAP2K4 and MAP2K7. Genes. Immun..

[195] Chandran P.A., Keller A., Weinmann L., Seida A.A., Braun M., Andreev K., Fischer B., Horn E., Schwinn S., Junker M. (2014). The TGF-beta-inducible miR-23a cluster attenuates IFN-gamma levels and antigen-specific cytotoxicity in human CD8(+) T cells. J. Leukoc. Biol..

[196] Xie N., Cui H., Banerjee S., Tan Z., Salomao R., Fu M., Abraham E., Thannickal V.J., Liu G. (2014). miR-27a regulates inflammatory response of macrophages by targeting IL-10. J. Immunol..

[197] Tang W., Yu F., Yao H., Cui X., Jiao Y., Lin L., Chen J., Yin D., Song E., Liu Q. (2014). miR-27a regulates endothelial differentiation of breast cancer stem like cells. Oncogene.

[198] GENECODE. https://www.gencodegenes.org.

[199] Klinge C.M. (2018). Non-Coding RNAs in Breast Cancer: Intracellular and Intercellular Communication. Noncoding RNA.

[200] Kong X., Liu W., Kong Y. (2018). Roles and expression profiles of long non-coding RNAs in triple-negative breast cancers. J. Cell Mol. Med..

[201] Balas M.M., Johnson A.M. (2018). Exploring the mechanisms behind long noncoding RNAs and cancer. Noncoding RNA Res..

[202] Pecero M.L., Salvador-Bofill J., Molina-Pinelo S. (2019). Long non-coding RNAs as monitoring tools and therapeutic targets in breast cancer. Cell Oncol. (Dordr.).

[203] Chen S., Zhu J., Wang F., Guan Z., Ge Y., Yang X., Cai J. (2017). LncRNAs and their role in cancer stem cells. Oncotarget.

[204] Huan J., Xing L., Lin Q., Xui H., Qin X. (2017). Long noncoding RNA CRNDE activates Wnt/beta-catenin signaling pathway through acting as a molecular sponge of microRNA-136 in human breast cancer. Am. J. Transl. Res..

[205] Zhang H., Cai K., Wang J., Wang X., Cheng K., Shi F., Jiang L., Zhang Y., Dou J. (2014). MiR-7, inhibited indirectly by lincRNA HOTAIR, directly inhibits SETDB1 and reverses the EMT of breast cancer stem cells by downregulating the STAT3 pathway. Stem Cells.

[206] Deng J., Yang M., Jiang R., An N., Wang X., Liu B. (2017). Long Non-Coding RNA HOTAIR Regulates the Proliferation, Self-Renewal Capacity, Tumor Formation and Migration of the Cancer Stem-Like Cell (CSC) Subpopulation Enriched from Breast Cancer Cells. PLoS ONE.

[207] Peng F., Li T.T., Wang K.L., Xiao G.Q., Wang J.H., Zhao H.D., Kang Z.J., Fan W.J., Zhu L.L., Li M. (2017). H19/let-7/LIN28 reciprocal negative regulatory circuit promotes breast cancer stem cell maintenance. Cell Death Dis..

[208] Peng F., Wang J.H., Fan W.J., Meng Y.T., Li M.M., Li T.T., Cui B., Wang H.F., Zhao Y., An F. (2018). Glycolysis gatekeeper PDK1 reprograms breast cancer stem cells under hypoxia. Oncogene.

[209] Lu G., Li Y., Ma Y., Lu J., Chen Y., Jiang Q., Qin Q., Zhao L., Huang Q., Luo Z. (2018). Long noncoding RNA LINC00511 contributes to breast cancer tumourigenesis and stemness by inducing the miR-185-3p/E2F1/Nanog axis. J. Exp. Clin. Cancer Res..

[210] Tu Z., Schmollerl J., Cuiffo B.G., Karnoub A.E. (2019). Microenvironmental Regulation of Long Noncoding RNA LINC01133 Promotes Cancer Stem Cell-Like Phenotypic Traits in Triple-Negative Breast Cancers. Stem Cells.

[211] Vidovic D., Huynh T.T., Konda P., Dean C., Cruickshank B.M., Sultan M., Coyle K.M., Gujar S., Marcato P. (2019). ALDH1A3-regulated long non-coding RNA NRAD1 is a potential novel target for triple-negative breast tumors and cancer stem cells. Cell Death Differ..

[212] Loewer S., Cabili M.N., Guttman M., Loh Y.H., Thomas K., Park I.H., Garber M., Curran M., Onder T., Agarwal S. (2010). Large intergenic non-coding RNA-RoR modulates reprogramming of human induced pluripotent stem cells. Nat. Genet..

[213] Wang Y., Xu Z., Jiang J., Xu C., Kang J., Xiao L., Wu M., Xiong J., Guo X., Liu H. (2013). Endogenous miRNA sponge lincRNA-RoR regulates Oct4, Nanog, and Sox2 in human embryonic stem cell self-renewal. Dev. Cell.

[214] Guttman M., Donaghey J., Carey B.W., Garber M., Grenier J.K., Munson G., Young G., Lucas A.B., Ach R., Bruhn L. (2011). lincRNAs act in the circuitry controlling pluripotency and differentiation. Nature.

[215] Shang M., Wang X., Zhang Y., Gao Z., Wang T., Liu R. (2018). LincRNA-ROR promotes metastasis and invasion of esophageal squamous cell carcinoma by regulating miR-145/FSCN1. Onco. Targets Ther..

[216] Lu P.W., Li L., Wang F., Gu Y.T. (2019). Inhibitory role of large intergenic noncoding RNA-ROR on tamoxifen resistance in the endocrine therapy of breast cancer by regulating the PI3K/Akt/mTOR signaling pathway. J. Cell Physiol..

[217] Eades G., Wolfson B., Zhang Y., Li Q., Yao Y., Zhou Q. (2015). lincRNA-RoR and miR-145 regulate invasion in triple-negative breast cancer via targeting ARF6. Mol. Cancer Res..

[218] Hou P., Zhao Y., Li Z., Yao R., Ma M., Gao Y., Zhao L., Zhang Y., Huang B., Lu J. (2014). LincRNA-ROR induces epithelial-to-mesenchymal transition and contributes to breast cancer tumorigenesis and metastasis. Cell Death Dis..

[219] Chen Y.M., Liu Y., Wei H.Y., Lv K.Z., Fu P. (2016). Linc-ROR induces epithelial-mesenchymal transition and contributes to drug resistance and invasion of breast cancer cells. Tumour. Biol..

[220] Hou L., Tu J., Cheng F., Yang H., Yu F., Wang M., Liu J., Fan J., Zhou G. (2018). Long noncoding RNA ROR promotes breast cancer by regulating the TGF-beta pathway. Cancer Cell Int..

[221] Zhang H.Y., Liang F., Zhang J.W., Wang F., Wang L., Kang X.G. (2017). Effects of long noncoding RNA-ROR on tamoxifen resistance of breast cancer cells by regulating microRNA-205. Cancer Chemother. Pharmacol..

[222] Li Y., Jiang B., Zhu H., Qu X., Zhao L., Tan Y., Jiang Y., Liao M., Wu X. (2017). Inhibition of long non-coding RNA ROR reverses resistance to Tamoxifen by inducing autophagy in breast cancer. Tumour. Biol..

[223] Zheng A., Song X., Zhang L., Zhao L., Mao X., Wei M., Jin F. (2019). Long non-coding RNA LUCAT1/miR-5582-3p/TCF7L2 axis regulates breast cancer stemness via Wnt/beta-catenin pathway. J. Exp. Clin. Cancer Res..

[224] Zhou M., Hou Y., Yang G., Zhang H., Tu G., Du Y.E., Wen S., Xu L., Tang X., Tang S. (2016). LncRNA-Hh Strengthen Cancer Stem Cells Generation in Twist-Positive Breast Cancer via Activation of Hedgehog Signaling Pathway. Stem Cells.

[225] Ma F., Liu X., Zhou S., Li W., Liu C., Chadwick M., Qian C. (2019). Long non-coding RNA FGF13-AS1 inhibits glycolysis and stemness properties of breast cancer cells through FGF13-AS1/IGF2BPs/Myc feedback loop. Cancer Lett..

[226] Keshavarz M., Asadi M.H. (2019). Long non-coding RNA ES1 controls the proliferation of breast cancer cells by regulating the Oct4/Sox2/miR-302 axis. FEBS J..

[227] Shin V.Y., Chen J., Cheuk I.W., Siu M.T., Ho C.W., Wang X., Jin H., Kwong A. (2019). Long non-coding RNA NEAT1 confers oncogenic role in triple-negative breast cancer through modulating chemoresistance and cancer stemness. Cell Death Dis..

[228] Youness R.A., Gad M.Z. (2019). Long non-coding RNAs: Functional regulatory players in breast cancer. Noncoding RNA Res..

[229] Hansji H., Leung E.Y., Baguley B.C., Finlay G.J., Askarian-Amiri M.E. (2014). Keeping abreast with long non-coding RNAs in mammary gland development and breast cancer. Front Genet..

[230] Li Z., Hou P., Fan D., Dong M., Ma M., Li H., Yao R., Li Y., Wang G., Geng P. (2017). The degradation of EZH2 mediated by lncRNA ANCR attenuated the invasion and metastasis of breast cancer. Cell Death Differ..

[231] Wu W., Chen F., Cui X., Yang L., Chen J., Zhao J., Huang D., Liu J., Yang L., Zeng J. (2018). LncRNA NKILA suppresses TGF-beta-induced epithelial-mesenchymal transition by blocking NF-kappaB signaling in breast cancer. Int. J. Cancer.

[232] Wang Z., Yang B., Zhang M., Guo W., Wu Z., Wang Y., Jia L., Li S., Xie W., The Cancer Genome Atlas Research Network (2018). lncRNA Epigenetic Landscape Analysis Identifies EPIC1 as an Oncogenic lncRNA that Interacts with MYC and Promotes Cell-Cycle Progression in Cancer. Cancer Cell.

[233] Barsyte-Lovejoy D., Lau S.K., Boutros P.C., Khosravi F., Jurisica I., Andrulis I.L., Tsao M.S., Penn L.Z. (2006). The c-Myc oncogene directly induces the H19 noncoding RNA by allele-specific binding to potentiate tumorigenesis. Cancer Res..

[234] Si X., Zang R., Zhang E., Liu Y., Shi X., Zhang E., Shao L., Li A., Yang N., Han X. (2016). LncRNA H19 confers chemoresistance in ERalpha-positive breast cancer through epigenetic silencing of the pro-apoptotic gene BIK. Oncotarget.

[235] Meseure D., Vacher S., Lallemand F., Alsibai K.D., Hatem R., Chemlali W., Nicolas A., De Koning L., Pasmant E., Callens C. (2016). Prognostic value of a newly identified MALAT1 alternatively spliced transcript in breast cancer. Br. J. Cancer.

[236] Cai Y., He J., Zhang D. (2016). Suppression of long non-coding RNA CCAT2 improves tamoxifen-resistant breast cancer cells’ response to tamoxifen. Mol. Biol. (Mosk.).

[237] Wu C., Luo J. (2016). Long Non-Coding RNA (lncRNA) Urothelial Carcinoma-Associated 1 (UCA1) Enhances Tamoxifen Resistance in Breast Cancer Cells via Inhibiting mTOR Signaling Pathway. Med. Sci. Monit..

[238] Saunders-Hastings P., Reisman J., Krewski D. (2016). Assessing the State of Knowledge Regarding the Effectiveness of Interventions to Contain Pandemic Influenza Transmission: A Systematic Review and Narrative Synthesis. PLoS ONE.

[239] Li W., Zhai L., Wang H., Liu C., Zhang J., Chen W., Wei Q. (2016). Downregulation of LncRNA GAS5 causes trastuzumab resistance in breast cancer. Oncotarget.

[240] Valadi H., Ekstrom K., Bossios A., Sjostrand M., Lee J.J., Lotvall J.O. (2007). Exosome-mediated transfer of mRNAs and microRNAs is a novel mechanism of genetic exchange between cells. Nat. Cell Biol..

[241] Wei Y., Lai X., Yu S., Chen S., Ma Y., Zhang Y., Li H., Zhu X., Yao L., Zhang J. (2014). Exosomal miR-221/222 enhances tamoxifen resistance in recipient ER-positive breast cancer cells. Breast Cancer Res. Treat..

[242] Yu D.D., Wu Y., Zhang X.H., Lv M.M., Chen W.X., Chen X., Yang S.J., Shen H., Zhong S.L., Tang J.H. (2016). Exosomes from adriamycin-resistant breast cancer cells transmit drug resistance partly by delivering miR-222. Tumour. Biol..

[243] Chen W.X., Cai Y.Q., Lv M.M., Chen L., Zhong S.L., Ma T.F., Zhao J.H., Tang J.H. (2014). Exosomes from docetaxel-resistant breast cancer cells alter chemosensitivity by delivering microRNAs. Tumour. Biol..

[244] Chen W.X., Liu X.M., Lv M.M., Chen L., Zhao J.H., Zhong S.L., Ji M.H., Hu Q., Luo Z., Wu J.Z. (2014). Exosomes from drug-resistant breast cancer cells transmit chemoresistance by a horizontal transfer of microRNAs. PLoS ONE.

[245] Mao L., Li J., Chen W.X., Cai Y.Q., Yu D.D., Zhong S.L., Zhao J.H., Zhou J.W., Tang J.H. (2016). Exosomes decrease sensitivity of breast cancer cells to adriamycin by delivering microRNAs. Tumour. Biol..

[246] Liu Q., Peng F., Chen J. (2019). The Role of Exosomal MicroRNAs in the Tumor Microenvironment of Breast Cancer. Int. J. Mol. Sci..

[247] Melo S.A., Sugimoto H., O’Connell J.T., Kato N., Villanueva A., Vidal A., Qiu L., Vitkin E., Perelman L.T., Melo C.A. (2014). Cancer exosomes perform cell-independent microRNA biogenesis and promote tumorigenesis. Cancer Cell.

[248] Vaupel P., Mayer A. (2007). Hypoxia in cancer: significance and impact on clinical outcome. Cancer Metastasis Rev..

[249] Hashimoto K., Ochi H., Sunamura S., Kosaka N., Mabuchi Y., Fukuda T., Yao K., Kanda H., Ae K., Okawa A. (2018). Cancer-secreted hsa-miR-940 induces an osteoblastic phenotype in the bone metastatic microenvironment via targeting ARHGAP1 and FAM134A. Proc. Natl. Acad Sci. USA.

[250] Turchinovich A., Samatov T.R., Tonevitsky A.G., Burwinkel B. (2013). Circulating miRNAs: cell-cell communication function?. Front Genet..

[251] Li M., Zhou Y., Xia T., Zhou X., Huang Z., Zhang H., Zhu W., Ding Q., Wang S. (2018). Circulating microRNAs from the miR-106a-363 cluster on chromosome X as novel diagnostic biomarkers for breast cancer. Breast Cancer Res. Treat..

[252] Eichelser C., Stuckrath I., Muller V., Milde-Langosch K., Wikman H., Pantel K., Schwarzenbach H. (2014). Increased serum levels of circulating exosomal microRNA-373 in receptor-negative breast cancer patients. Oncotarget.

[253] Kong X., Zhang J., Li J., Shao J., Fang L. (2018). MiR-130a-3p inhibits migration and invasion by regulating RAB5B in human breast cancer stem cell-like cells. Biochem. Biophys. Res. Commun..

[254] Chen W.X., Cheng L., Pan M., Qian Q., Zhu Y.L., Xu L.Y., Ding Q. (2018). D Rhamnose beta-Hederin against human breast cancer by reducing tumor-derived exosomes. Oncol Lett..

[255] Jang J.Y., Lee J.K., Jeon Y.K., Kim C.W. (2013). Exosome derived from epigallocatechin gallate treated breast cancer cells suppresses tumor growth by inhibiting tumor-associated macrophage infiltration and M2 polarization. BMC Cancer.

[256] Zhang J., Zhang H.D., Yao Y.F., Zhong S.L., Zhao J.H., Tang J.H. (2015). beta-Elemene Reverses Chemoresistance of Breast Cancer Cells by Reducing Resistance Transmission via Exosomes. Cell Physiol. Biochem..

[257] Wei Y., Li M., Cui S., Wang D., Zhang C.Y., Zen K., Li L. (2016). Shikonin Inhibits the Proliferation of Human Breast Cancer Cells by Reducing Tumor-Derived Exosomes. Molecules.

[258] Hannafon B.N., Carpenter K.J., Berry W.L., Janknecht R., Dooley W.C., Ding W.Q. (2015). Exosome-mediated microRNA signaling from breast cancer cells is altered by the anti-angiogenesis agent docosahexaenoic acid (DHA). Mol. Cancer.

[259] O’Brien K.P., Khan S., Gilligan K.E., Zafar H., Lalor P., Glynn C., O’Flatharta C., Ingoldsby H., Dockery P., De Bhulbh A. (2018). Employing mesenchymal stem cells to support tumor-targeted delivery of extracellular vesicle (EV)-encapsulated microRNA-379. Oncogene.

[260] Bliss S.A., Sinha G., Sandiford O.A., Williams L.M., Engelberth D.J., Guiro K., Isenalumhe L.L., Greco S.J., Ayer S., Bryan M. (2016). Mesenchymal Stem Cell-Derived Exosomes Stimulate Cycling Quiescence and Early Breast Cancer Dormancy in Bone Marrow. Cancer Res..

[261] Roma-Rodrigues C., Pereira F., Alves de Matos A.P., Fernandes M., Baptista P.V., Fernandes A.R. (2017). Smuggling gold nanoparticles across cell types—A new role for exosomes in gene silencing. Nanomedicine.

[262] Naseri Z., Oskuee R.K., Jaafari M.R., Forouzandeh Moghadam M. (2018). Exosome-mediated delivery of functionally active miRNA-142-3p inhibitor reduces tumorigenicity of breast cancer in vitro and in vivo. Int. J. Nanomedicine.

[263] Jin H., Yu Y., Chrisler W.B., Xiong Y., Hu D., Lei C. (2012). Delivery of MicroRNA-10b with Polylysine Nanoparticles for Inhibition of Breast Cancer Cell Wound Healing. Breast Cancer (Auckl.).

[264] Devulapally R., Sekar N.M., Sekar T.V., Foygel K., Massoud T.F., Willmann J.K., Paulmurugan R. (2015). Polymer nanoparticles mediated codelivery of antimiR-10b and antimiR-21 for achieving triple negative breast cancer therapy. ACS Nano..

[265] Deng X., Cao M., Zhang J., Hu K., Yin Z., Zhou Z., Xiao X., Yang Y., Sheng W., Wu Y. (2014). Hyaluronic acid-chitosan nanoparticles for co-delivery of MiR-34a and doxorubicin in therapy against triple negative breast cancer. Biomaterials.

[266] Ekin A., Karatas O.F., Culha M., Ozen M. (2014). Designing a gold nanoparticle-based nanocarrier for microRNA transfection into the prostate and breast cancer cells. J. Gene Med..

[267] Zhi F., Dong H., Jia X., Guo W., Lu H., Yang Y., Ju H., Zhang X., Hu Y. (2013). Functionalized graphene oxide mediated adriamycin delivery and miR-21 gene silencing to overcome tumor multidrug resistance in vitro. PLoS ONE.

[268] Hydbring P., Wang Y., Fassl A., Li X., Matia V., Otto T., Choi Y.J., Sweeney K.E., Suski J.M., Yin H. (2017). Cell-Cycle-Targeting MicroRNAs as Therapeutic Tools against Refractory Cancers. Cancer Cell.

